# Pharmacy, Testing, and the Language of Truth in Renaissance Italy

**DOI:** 10.1353/bhm.2017.0026

**Published:** 2017

**Authors:** Valentina Pugliano

**Keywords:** drug testing, drug adulteration, natural history, Italian pharmacy, materia medica, botanical terminology, taxonomy, authenticity, apothecaries, artisans

## Abstract

This article examines the role of testing and innovation in sixteenthcentury Italian pharmacy. I argue that apothecaries were less concerned with testing drugs for efficacy or creating novel products than with reactivating an older Mediterranean pharmacological tradition and studying the materials on which it relied. Their practice was not driven by radical experimentation but by a "culture of tweaking"—of minute operational changes to existing recipes and accommodation of their textual variants—which was rooted in the guild economy fostering incremental over radical innovation and in a humanist reevaluation of past autorities. Workshop practice was also increasingly driven by a new ideal of staying true to nature fostered by the period's botanical renaissance. This led to an emphasis on ingredients over processes in the shop, and found clearest expression in the elaboration of a taxonomic "language of truth" that helped apothecaries discern between authentic and inauthentic *materia medica* and harness their sincerity in lieu of testing effectiveness.

*Probatum est*. It has been tried and proven to work. Adorning countless recipe books and the scattered prose of craftsmen, this pithy sentence has come to encapsulate the view that modern historians hold of artisanal practice in medieval and early modern Europe—one of inquisitiveness and flexible learning, of testing matter and testing hypotheses by working[Other P-233] through matter.^1^ Drug making is often considered a case in point. The sixteenth and seventeenth centuries witnessed the production and aggressive marketing of multifarious remedies, as health products became a goal in sites as diverse as aristocratic kitchens and monastic infirmaries. Some were novel creations, notably chemical remedies. Others were revised versions of old favorites. Most needed to gain the trust of increasingly discerning consumers.^2^ Yet, we also know better than to take the *probatum est* claim at face value. Some practitioners lied about their experiences and results. Others were content to rely on the feats of third parties, whether acquaintances or famous doctors, who vouched for a certain preparation and its effects.^3^ In this article, I explore this tension around experimentation in workshop practice through the case of Italian institutional pharmacy, a medical context examined so far only fragmentarily. To what extent did drug testing actually take place in the average pharmacy of Renaissance Italy? What purposes did it serve? And how was testing evaluated against other modes of validating opinions and operative choices in the shop?

*Speziali da medicina*, or apothecaries, constituted a distinctive and populous group in the medical landscape of early modern Italy. Like surgeons (*cirugici*) and barbers (*barbieri*), they had been organized into guilds (*arti*) and regulated through statutes by the civic authorities since the thirteenth century. By the sixteenth, larger centers like Venice, Florence, and Rome counted dozens of masters, apprentices, and journeymen who operated from dedicated shops, stocked at great expense, and maintained close contacts with the merchant network at home and in the Levant's spice emporia. They dispensed to the public, and served their city by supplying hospitals and pesthouses in times of crisis and by lending their expertise on poisons in court trials and, occasionally, in more sinister political schemes. To exercise their art, these practitioners were required to pass an examination at the end of several years of training that included the acquisition of basic Latin literacy. As a group, they were closely associated[Other P-234] with the Hippocratic-Galenic tradition of pharmacology, and had both family ties and a tight working relationship with university-educated physicians and their learned culture.^4^ These factors ensured that apothecaries, while often depicted as encroached upon by freelancing charlatans and empirics,^5^ held the monopoly over the preparation and retail of drugs across the peninsula throughout the early modern period.^6^

The current reappraisal of the artisanal world and its creativity around making things has both engendered a productive dialogue between historians of medicine and science and helped revise traditional narratives about the rise of an experimental culture, reinserting artisans among its rightful protagonists.^7^ Apothecaries undoubtedly belong to this story. I believe, however, that the definitions of testing and trying that have been central to this historiographical reappraisal do not capture adequately the distinctiveness of the medical trades. In this article, I set out to interrogate such definitions by examining the role of testing in pharmacy and by inquiring how experimentation, inquisitiveness and innovation were actually perceived by the apothecaries themselves.

The focus on institutional pharmacy presents us immediately with two methodological challenges. First, we should carefully draw boundaries (however porous) between trade and craft contexts and contexts where, on the other hand, experimentation featured as an intellectual endeavor with no immediate bearing on the individual's livelihood. Here, I adopt Ursula Klein and Emma Spary's caution about addressing materials and products originating in commercial contexts. While these could and often[Other P-235] did intersect with the spaces and themes of learned inquiry (especially so for drugs in sixteenth-century Italy, where most seekers of natural knowledge had a medical background), they were ultimately "object[s]-in-the-world," everyday items meant to be a source of profit. They were engendered and operated in a setting where, as Klein and Spary point out, notions of philosophical experiments, trialling and replication, though present in some cases and to some degree, were clearly not center stage.^8^ This duality should be especially highlighted for the goods produced by apothecaries and similar medical artisans practicing formally within the structures of corporations. Their remedies were first of all utilitarian, retail objects manufactured within set institutional guidelines which only under certain circumstances and in some hands became sources of curious inquiry. Second, while pharmacy was a commercial endeavor, it is important to remember its peculiarity. The need to restore health, the fragility of the human body, and the subjectivity of response to treatment ensured that apothecaries worked under different goals and constraints from those of other craftsmen—be they goldsmiths or shoemakers—and followed a different pace of inquiry and technological change. Experimentation, in other words, has a plural history also among the crafts. This article goes toward recovering a facet of this plurality. More broadly, by addressing these concerns, it adopts a somewhat polemical stance on the disciplinary dynamic between the histories of science and medicine, warning against assuming a seamless transfer of concerns from one to the other.^9^ While medicine should be considered an integral contributor to the long history of experiment, the specificity of its contribution must remain in the foreground.

In this article I argue first that, while there was a dynamic engagement with ingredients and methods in the sixteenth-century pharmacy shop, drug testing was itself limited. Experimentation served more often to work through the small obstacles, material and textual, encountered in everyday practice than as a means to assess the effectiveness and worth of the remedies thus produced. Ultimately, I argue that the epistemological focus in the workshop moved away from techniques to materials, and the[Other P-236] identification of ingredients came to hold more significance and visibility than processes of production. The clearest symptom of this was the elaboration of what I call a "language of truth" to navigate the sea of rare and misidentified simples arriving in the shop. Its taxonomy of sincerity and falsehood captured a particular moment in the intellectual history of European medicine and science. It was a direct response to both the traditional problem of drug adulteration, and the methodological crisis precipitated in the late fifteenth century by the recovery of the ancients' *materia medica* and the birth of medical botany. It also reflected Italian pharmacy's growing intellectual investment in that ideal of antiquity and faithfulness to textual scholarship that humanism nurtured among all categories of medical practitioners across Italy.^10^ For those complex compounds such as Theriac that gave Renaissance Italian pharmacy its name, testing was easily supplanted by this "rhetoric of truth" that equated the use of correct, authentic ingredients with the recipe's efficacy.

## Testing

A cursory survey of the writings left by sixteenth-century Italian apothecaries, from personal letters to polemical pamphlets, yields several instances of the terms "experience" (*esperientia*) and "experiment" (*esperimento*) to denote both the general knowledge acquired from daily toil and exposure to recurrent problems in the *bottega*, and specific instances where the eyewitnessing of a phenomenon imparted particular usable information.^11^ This terminology was part of a larger endorsement of practice and the sensorium as sources of knowledge, through which this artisan group vindicated the worth of the firsthand engagement with materials that provided its livelihood and status.^12^ It also reflected a new positive attitude toward empiricism among the scholars of the period.^13^

Yet, a close analysis of the apothecaries' trade literature does not bear out a culture of testing in the workshop—at least if we take testing to be a means to establish the *efficacy* of the drugs on sale and to determine which preparations should be produced and which should not; or,[Other P-237] methodologically, to be a structured and repeated process of setting out a question or hypothesis in advance, estimating results, and seeking evidence in support or refutation in the manner of seventeenth-century learned drug trials.^14^ Terms that may be associated with such methodology, *prova* and *provare*, occur rarely and mostly in relation to occasional trials with poisons and their antidotes. Nor are batches of remedies usually tried out to verify their powers before administration. If queried at all, efficacy is established by other means: by invoking tradition and literary precedent, or by turning to trustworthy peers and their anecdotal evidence. This is not to say that creative experimentation with substances had no place in the Renaissance pharmacy, but it was left to individual curiosity and was usually triggered by an interest in discourses tangential to healing, notably natural history and metallurgical alchemy.^15^ Where *everyday trade* was concerned, I contend that the average Italian apothecary neither tested whole drugs nor tried cures.

What one could observe across the shops was a "culture of tweaking": a low-humming, steady level of activity probing materials, honing methods, and applying small revisions to recipes. This work of tweaking, however, was meant neither to reassess canonical products, nor to yield new remedies. It also relied, as often as not, on insights into the uses of materials obtained serendipitously. Rather, it was a modus operandi rooted in the long tradition and strict regulations of the apothecaries' art, which allowed for recipes to be adjusted but rarely substantially altered.^16^

### Tweaking Traditional Recipes

The most vocal sources on workshop practice are found in a new genre of descriptive pharmacopoeias authored by apothecaries and the odd[Other P-238] physician of north and central Italy in the second half of the sixteenth century: Girolamo Calestani of Parma's *Delle osservationi nel comporre gli antidoti e medicamenti chepiù si costumano in Italia all'uso della medicina* (1562); *La fabrica degli spetiali* (1566) by the Paduan professor of anatomy Prospero Borgarucci; the *Avvertimenti nelle composition per uso della spetiaria* (1575) by the German-born and Venice-based Giorgio Melichio; and the Mantuan Filippo Costa's *Discorsi sopra le composition degli antidoti & medicamenti che più si costumano di dar per bocca* (1576).^17^ In affordable quarto format and in the vernacular, these texts encountered immediate popularity. The works of Calestani and Melichio especially saw dozens of editions. Calestani's *Osservationi* was eventually adopted as the official antidotary of the Duchy of Parma and Piacenza in 1667, while Melichio's *Avvertimenti* was translated into Latin in 1586 and continued to be enlarged by his successors at the Ostrich pharmacy Paolo Romani, Alberto Stecchini, and Antonio De Sgobbis, who gradually incorporated into it chemical medicine.^18^ This corpus of texts inaugurated a new style of discussion of recipes that continued to be emulated (and plagiarized) by fellow practitioners throughout the seventeenth century and across the length of the peninsula, beginning with the *Ricettario* (1604) of Giuseppe Santini of Lucca and the *Discorsi* (1625) of the Sicilian Salvatore Francioni.^19^

Indeed next to short essays on Theriac and its ingredients, descriptive pharmacopoeias constitute the main genre of trade literature published by early modern Italian apothecaries, and signal the culmination of the professionalization of the trade begun in the fifteenth century.^20^[Other P-239] Their success, as we shall see, was tied to their novel approach to the textual transmission of practice. While they continue the rich tradition of pharmacological writing developed around the ancient and medieval Mediterranean basin, in fact, they differ markedly from the two other dominant subgenres available in medieval and early modern Europe: herbals and their A-to-Z descriptions of simples; and civic antidotaries and shop formularies (*pandette*) that provided prescriptive lists of ingredients and quantities, such as Florence's official pharmacopoeia, the *Ricettario Fiorentino* (1498, 1550).^21^ The descriptive pharmacopoeias run instead to hundreds of narrative pages where apothecaries do not simply review recipes, but also advertise their knowledge, vent and describe their struggles with remedy preparation, examine extensively the opinions held in canonical books, tell anecdotes, praise friends and scorn colleagues, and heartily plagiarize each other. The dedications, indices of cited authors, and internal references testify to their authors' links with local academic circles and the new community of naturalists. Personal acquaintances include Pietro Andrea Mattioli, the curator of Padua University's botanic garden Giacom'Antonio Cortuso, and the Neapolitan polymath Vincenzo Pinelli. The manuals, however, are intended primarily for fellow artisans, apprentices, and practicing physicians (customarily attached to one or more shops). Crucially, while certainly conceived as tools of self-promotion, the descriptive pharmacopoeias do not aim to propose the author's proprietary medicines in the manner of books of secrets and marketing pamphlets, but to survey the corpus of remedies habitually prepared in the shops of the peninsula.^22^ Part of their unique documentary value lies precisely in their serendipitous recording of the work of neighboring practitioners and the therapeutic preferences of certain towns.

What these texts extensively document is the practice of tweaking existing recipes. Such tweaking and tinkering could concern any aspect of preparation: ingredients, processes, and equipment. For instance, instructing on the best way to prepare simples for the famous Theriac, Giorgio Melichio discouraged his readers from following Galen's directions on one[Other P-240] point. Instead of grinding together ingredients of different consistencies like roots and petals, one should tackle the more time-consuming hard items first, then the semi-hard, then the soft in separate mortars: "The experiment found a better way … than the one written by the Ancients and this method apothecaries retain, trusting more in the Art's general rules and experience than in Galen's authority, despite the latter being great."^23^ Melichio also recommended exposing rose and crocus flowers to the sun rather than exsiccating them before a fire, "as most do," before crushing them.^24^ Testifying to these pharmacopoeias' intertextuality, and to the apothecaries' alertness to each other's authorial decisions, in his own manual Filippo Costa praised his Neapolitan colleague Ferrante Imperato for adopting this variant of practice.^25^

Other instances of tweaking concerned the sequence of passages, such as preparing one class of ingredients before another; timing, such as the number of hours to rest the ingredients before their final mixing; operations, such as varying the liquids used as facilitators for mixing; equipment, such as the advantages of a stone mortar over an iron one; and the range of ingredients, such as substituting a secondary ingredient to prolong a product's shelf life. Thus, reviewing Rhasis's recipe for Pills against the Plague, Prospero Borgarucci approved the choice "of some" of replacing wine with syrup of lemon or citron, as "they conserve the mass for longer."^26^ Melichio, instead, refused to dip Oriental anacardium in vinegar "as some do following Manlio," because his infusion of the same had kept so well without resorting to this step that he was able to use it after two years for a friar from St Stephen's convent in Venice with the blessing of his attending physician.^27^ Experience was often cited as the source of learning that, by introducing or withholding certain changes, the preparation would be smoother and the compound's quality improve as a result. Once verified, such procedural changes were usually applied across the board to remedies requiring the same preparatory steps.

Here, tweaking does not simply denote the intrinsic suppleness of the recipe as a verbalized format of practice, that flexibility demanded of anyone wishing to put a set of written instructions into practice. It does not describe, in other words, those small adjustments that are not[Other P-241] programmatic but are often applied impromptu, in reaction to an assessment of the specific circumstances encountered during preparation—such as leaving the proverbial chicken in the oven for an additional five minutes.^28^ There are certainly glimpses of such suppleness in the pharmacopoeias. For example, discussing the general rule (*regola*) for preparing syrups—adding a pound and a half of juice or infusion for each pound of honey or sugar—Costa notes, "in some cases we have not observed it so minutely, either because of the delicacy or nature of the liquors involved."^29^

The tweaking I speak of was more substantive. It was a work of adjustment triggered by the need to accommodate separate bodies of knowledge—frequently referred to by the apothecaries with the expression "ancients and moderns" (*antichi et moderni*)—which the editorial activities of humanism and the increased authorship of medical practitioners had mobilized and unsettled.^30^ New recipes and information on ingredients had become available thanks to the sixteenth-century editions of Greco-Roman treatises on *materia medica* (particularly those by Pliny and Dioscorides), and the new writings on practical medicine and surgery by authors like Bartolomeo Montagnana (fl. 1422–60) and Giovanni da Vigo (1450–1525). It was these texts that shop physicians increasingly consulted to write their prescriptions. It was these texts that apothecaries had now to reconcile with the medieval Arabic and Latin *auctoritates* that still held sway in the workshop (notably Pseudo-Mesue/Ibn Masawayh, Rhasis/al-Razi, and the Salernitan School's *Antidotarium Nicolai*),^31^ and with the practical tradition embodied by magisterial remedies (*rimedi magistrali*). The latter were remedies that had been devised by often nameless masters and that in the sixteenth century circulated with a consolidated recipe primarily via the manuals of apothecaries Giacomo Manlio's *Luminare majus* (1494) and Paulo Suardo's *Thesaurus aromatariorum* (1496), precursors of the descriptive pharmacopoeias.

Tweaking helped to solve the plenitude of competing and conflicting advice that these different sources had made available on the same remedy. Wishing to prepare Mesue's Trociscks of Alandahal, Girolamo Calestani reportedly found himself with some texts prescribing ten ounces and[Other P-242] others ten drams of coloquintida, "possibly from an error of the interpreters or the printers." Thus, "in order to know the best and truest [dosage] we prepared it both ways, and always found the first to be better, being less oily and not so weak in coloquintida as the second."^32^ The challenges of making sense of a translated tradition were constitutive of navigating the debate of ancients and moderns. Commenting on Nicolò Fiorentino's recipe for Syrup of Chicory, Costa described his predecessors' difficulties in establishing the correct ingredients from the available scripts: "back then some put *cucúrbita* [pumpkin] in place of *cicerbita* [blue sow thistle], and others *lupini* [lupins] in place of *lupoli* [hops]."^33^ Calestani plainly chastised his contemporaries' lack of historical acumen, as they failed to realize that charcoal existed in Mesue's time and thus misunderstood his instructions on the Electuary of Rosato by cooking scammony on too strong a flame stoked with dry wood.^34^ Ultimately, discrepancies in the written evidence were assessed with a "workshop judgment" cultivated through long familiarity with the materials of the craft and the challenges posed by their manipulation.^35^

**Figure 1:**
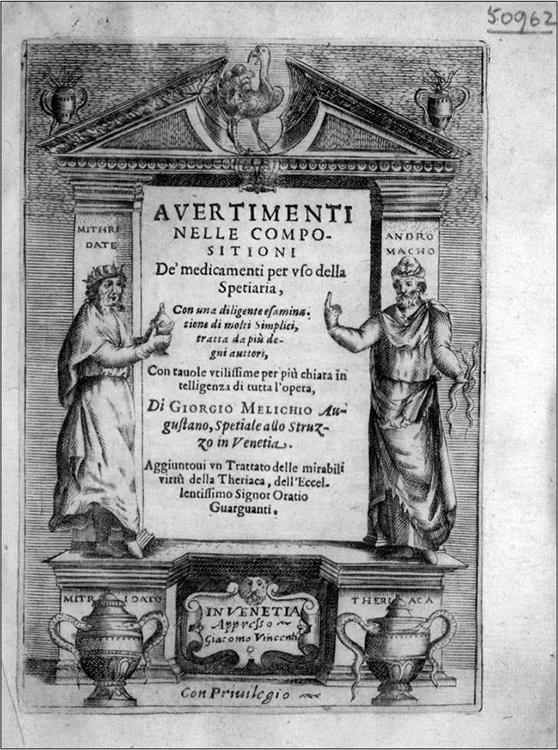
Frontispiece of Giorgio Melichio, *Avvertimenti nelle compositioni per uso della spetiaria* (Venice, 1605), adapted from an existing design shared among Venetian print shops. Courtesy of the Wellcome Library, London.

This recourse to practice to ameliorate and solve awkward points in the execution of recipes resembles what Pamela Smith recently described as "working through resistances of matter," a process of learning through obstacles and failures that for most craftsmen would have been, and still is, intrinsic to finding the correct pathways to making things.^36^ Yet, an even better analogy would be that between the apothecary and the modern historian engaging in reconstruction from how-to books, like Smith herself. The authors of Renaissance pharmacopoeias present themselves as both creators and reenactors, struggling through the obscurity and idiosyncrasies of an enlarged canon to replicate and render once again viable the recipes of their predecessors.^37^ This awareness surfaces[Other P-243] through antiquarian metaphors and references to originals waiting to be uncovered that resonate with a wider humanist discourse advocating the intellectual and civic renewal of society by return to an idealized past.^38^ So, for Costa a recipe needs to be restored (*restaurare*), almost as one would piece together a ruin; for Prospero Borgarucci it is "brought back to light."^39^ It is an ambition that finds its ultimate emblem in the frontispiece of Melichio's pharmacopoeia, which asks the reader to walk through the Ostrich pharmacy's imaginary shop front supported by two pillars of ancient pharmacological knowledge, Mithridates VI King of Pontus (first century BC) and Andromachus the Elder (first century AD), the legendary creators of the antidotes Mithridate and Theriac (see Figure 1).

**Figure 2:**
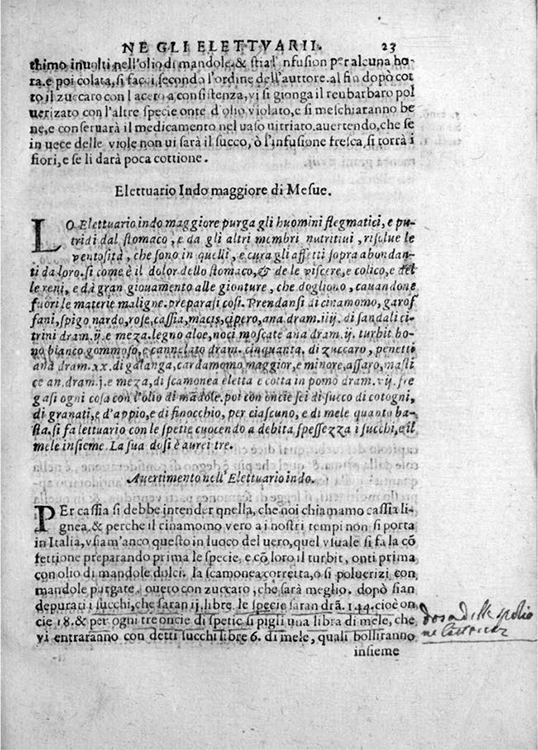
Scholion for the recipe of the "Indo Maggiore"—a mixture of observations, warnings, and advice as its heading "awertimento" suggests—from Giorgio Melichio, *Avvertimenti nelle compositioni per uso della spetiaria* (Venice, 1605), sig. 23r. Courtesy of the Wellcome Library, London.

That the challenges were perceived as being not only of materiality, but of textual disambiguation is reflected in turn in the prominence given to glossing in the descriptive pharmacopoeias. Each recipe, whether attributed to a past luminary or to the apothecaries' collective magisterial tradition, is followed by a longer authorial scholion, frequently demarcated on the page by the subheading *osservatione* or *avvertimento*, where authors are weighed against each other, passages explained, and personal experiences recounted (Figure 2). Girolamo Calestani explicitly identified these observations as the innovative element of his manual, otherwise concerned with the recipes of his predecessors, because "many things have been left without interpretation or have been interpreted obscurely … and no writing (excluding the divine), however good, requires no correction, improvement or limitation."^40^ By adopting this commentary mode, apothecaries inserted themselves in an established heuristic tradition of academic medicine, lending gravitas to their authorial voice.^41^ More importantly, they succeeded in producing manuals that reconciled interrelated but separate traditions and customs of practice, and codified a way forward. The way forward consisted in a curated assemblage of positions that left room for individual reasoning.

This work of streamlining is particularly important when we consider that the artisans' descriptive pharmacopoeias predate most of the official antidotaries written by university-educated physicians that the Colleges of Physicians of cities like Bologna, Bergamo, Rome, and Naples began to commission in the late sixteenth century and early seventeenth precisely[Other P-244] to solve confusion and standardize local practice.^42^ They also went against their prescriptive spirit. This revealing aside comes from the 1639 manual of the Neapolitan Friar Donato:

[Other P-245]

[Other P-246]

One shouldn't laugh at the apothecary who wants, following his experience, his Art, his reason, and the authority of knowledgeable Physicians, to correct some recipe of the Ancients, written in an obscure manner; as one should laugh (as we do every day) of the many prescriptions ordered by famous doctors, which have no rule, no method and no art and cannot be prepared unless the apothecary corrects them with his judgement.^43^

Friar Donato was castigating Curzio Marinelli, notorious author of the first civic pharmacopoeia of Venice (1617), which had to be withdrawn from the market almost immediately for the scathing comments against apothecaries it contained.^44^ Yet, beside the prejudice informing medical hierarchies in the period, it is not difficult to see how tweaking may have been perceived as undermining the prerogative of prescribing traditionally claimed by the physicians.^45^

As archives of standards and variants, these descriptive pharmacopoeias were probably not meant for step-by-step use in the manner of standard formularies. Indeed they did not always spell out their recipes nor indicate ingredients' quantities, relying instead on other manuals to provide such information (a useful reminder that some categories of artisans worked in and from a very literate environment). Rather, they served for doublechecking the complications associated with certain procedural choices and the validity of others. Their format invited active questioning, further reading, but also further practice. That they were used as working manuals is testified not only by their editorial fortune, but also by their presence on the shelves of Italian pharmacy shops, whose probate inventories record editions of "Callestani and Melliquio" alongside copies of Mesue and Mattioli's *Dioscorides*.^46^ The master apothecary achieved self-promotion precisely by taking these instructions for tweaking outside the realm of tacit knowledge, and the opinions and choices put forward continued to be carefully weighed by his seventeenth-century successors.^47^[Other P-247]

In turn, tweaking served him as an important proprietary mechanism also outside the written page. As an operative tool, it gave apothecaries room to express their individuality and leave their magisterial mark on standard products in a very competitive market, without stepping away from a written tradition that was held in high regard by both artisans and their closest partners, physicians. Interspersed throughout the pharmacopoeias are references to the "rules" followed in the shops of fellow masters.^48^ The manuals point to this knowledge of variant practices circulating on paper, but also orally, discussed during guild meetings and visits to colleagues, in technological exchanges that benefited from both the custom of clustering shops along a city's thoroughfares like the Rialto in Venice, and the peripatetic education of young apothecaries who often left home to train in larger market towns.^49^ Giorgio Melichio intentionally recorded Venetian customs that would otherwise be lost: "It is common in the pharmacies of Venice to employ Diacodion [a cordial] in its solid form, yet because no author describes this manner of preparation, I will now write as much as can be observed in the shops of good and expert apothecaries."^50^ Similarly, Calestani, who in his *Osservationi* frequently reports the punctual operational advice heard as an apprentice in Rome from the friars of the Aracoeli monastery in Campidoglio, also related the recipe for a magisterial lenitive electuary, "a truly beautiful composition," shown to him later in life by colleagues in Mantua, "and by them solely observed."^51^

At this point, it is important to stress that the micro-testing involved in tweaking does not contradict the general methods of preparation of common compounds, nor does it ever result in a significant alteration of the recipe. (What Melichio's colleagues prepared in Venice, albeit with a twist, was still recognized as Diacodion by their peers across Italy.) The changes introduced at the individual's discretion, often within a simple binary choice offered by existing manuals, are always small in scale, and the overall procedure remains fairly stable. The resulting compound would not be, or be considered, substantially different. It would also be administered to patients with little fanfare and certainly without prior trial.

The absence of thorough testing for efficacy of the existing pool of remedies can be partly explained by the degree of flexibility already built into pharmaceutical practice, which prescribed adjusting drugs to individual[Other P-248] needs.^52^ Primarily, however, it reflects the fact that effectiveness was not at stake in this setting. Tweaking was not designed to recuse a pharmacological tradition. To the contrary, tweaking concerned tiny improvements over a palimpsest that in principle was already believed to work.

One central reason for this palimpsest's resilience was the importance accorded to tradition and custom. Artisans were understandably reluctant to modify established routines of production that had proved efficient and profitable.^53^ The apothecaries' pharmacopoeias mention frequently the lesson provided by the rules of the art (*regole dell'arte*):^54^ those consolidated ways of doing things shared by all the practitioners of a craft, a common knowledge taught through apprenticeship and sanctioned by the examination that marked the apothecary's official entry into the guild. The artisan, moreover, had to negotiate external constraints. The oversight exercised by the guild varied according to town and circumstances, though, generally, a high degree of uniformity was expected, especially for complex "named compounds," which were inspected annually.^55^ Similarly, the local College of Physicians played its role, as apothecaries were statutorily forbidden from dispensing internal and compound remedies without a doctor's prescription. "Because I am its member"—so Costa justified following the recipe for Syrup of Endive given in the official antidotary of "our College of Mantua."^56^

Tradition also manifested itself as respect for and reliance on the work of predecessors, both ancient and medieval. We have seen Italian pharmacy's textual nature. It would be a slight, according to Melichio, "to pervert the medicament against the author's intent, without authority or a reason of any importance."^57^ He thus accused his colleague Calestani of exercising unduly his "invention and imagination" for using the syrup of violet and aniseed instead of the raw ingredients in the famous Diacatholicon of Nicholas of Salerno—in other words, for tweaking.^58^ This was not just rhetorical posturing. In fact, this "conservatism" traced part of its roots[Other P-249] to an earlier, prestigious tradition of practice that had addressed similar problems of drug accreditation. As Michael McVaugh has pointed out, the reintroduction in thirteenth-century European universities of Greco-Arabic pharmacology, and later the New Galen, not only entailed substantial adjustments to the textual canon, but also raised concerns around classes of remedies that would become staples of early modern pharmacy: laxatives, opiates, and caustics. Their potency, believed to have the potential to quickly turn into harm, ensured that they were scrutinized closely.^59^ By the sixteenth century, in other words, apothecaries could look back to a consolidated tradition of drug "testing" and skepticism, of stabilization of compounds and therapeutic choices, which exonerated them from the need of more radical endeavors.

These factors do not mean that practice was static and unchanging—far from it. But they require us to frame our questions differently and account more closely for the intellectual horizon in which these artisans placed their practice.

### Trying Secrets and Novelty

Central to the issue of limited modification and testing of common drugs are larger questions about the place of novelty in Renaissance pharmacy and the apothecaries' understanding of notions of improvement and perfectibility in their art, which here can be raised only briefly. The problem of innovation has always loomed large for historians of science and technology and has often been tied to forms of experimentation.^60^ The recent material turn, while widening the focus from the libraries of natural philosophers to the workshops of common craftsmen, has made innovation almost synonymous with the artisanal world, often without problematizing this association.^61^ For their part, historians of early modern medicine seem to vacillate between an interest in the discontinuities and, more recently, the continuities of the *ars longa* across the periods.^62^[Other P-250]

The emendation and rehabilitation of Greco-Roman *materia medica* were probably the most important innovations to occur in late fifteenth and sixteenth-century Italian institutional pharmacy.^63^ The practice of tweaking documented in the descriptive pharmacopoeias provides an insight into how this tradition was absorbed in workshop practice. It also supports the view of a new generation of economic historians like Stephan Epstein who emphasize how, far from being stagnant institutions, guilds nurtured an "environment for technical change," not through radical breaks from established practice but through "small-scale and incremental practical experiment and random variation."^64^ As it streamlined those choices of practice that had been proven to be more successful than others, tweaking provides a model of how such small-scale variation and innovation, which often remained in the realm of tacit knowledge, occurred and what drove them.

The nature of this innovation, and the artisans' attitude toward it, need, however, to be taken into account. In craft work, changes were made primarily as need arose. The notion of drug experimentation for modern and contemporary scientific and medical cultures may be said to be strongly future-oriented and openly promote an idea of improvement that is only partially borne out by sixteenth-century pharmaceutical literature.^65^ Though apothecaries may state a concern not to produce a remedy with weak or no effect, and may motivate testing with the need to avoid mix-ups and unwillingly harming patients, we should be careful about viewing their activities as driven by an abstract idea of better care. Nor do they subscribe to the notion of employing radical testing or experimenting to create entirely new products, in this differing from proponents of medical alchemy and iatrochemistry who made of their[Other P-251] ability to transfigure materials a selling point and key feature of their identity as knowers of nature.^66^

Overall practitioners of institutional pharmacy displayed a different understanding of innovation, one that dissociated novelty from technical progress and placed the latter in a nonlinear conception of time.^67^ Their sixteenth-century pharmacopoeias praised the classical past and discussed remedies that had accumulated in the previous three centuries. On one hand, advancing knowledge meant reinstating this preexisting yet lost corpus and integrating it with the current one. The manuals indirectly capture a sense of progression with remarks like that of Borgarucci on the newly discovered White Eyedrops of Rhasis—"At the time of Manlio [late fifteenth century] this collyrium was not used," remarks that reveal how the apothecaries saw their practice as dynamic and perfectible.^68^ On the other hand, the impression is that the practitioners of institutional pharmacy still worked within the framework inherited from scholastic writings on pharmacy, and scholastic medicine's general attitude toward the "progressive growth of knowledge." The system, as Chiara Crisciani has stressed, allowed for dynamism and for the absorption of seemingly extraneous and contradictory knowledge, but itself was ultimately closed.^69^ Accordingly, tweaking did not produce new experimental goals. It made the existing system work.

Indeed, rather than being a premium, novelty was seen with suspicion. It was one reason for the limited therapeutic use of American plants like guaiacum, china root, and sarsaparilla beyond the treatment of morbus gallicum, itself exceptional in that it was believed to be a "new" disease.^70^[Other P-252] As late as 1573 the possibility of replacing the rare Oriental balsam required for Theriac with a liquid from the Indies provoked a controversy among Florentine apothecaries.^71^ The fact that American imports remained exiguous throughout the sixteenth century only contributed to the resilience of this discourse. Similarly, novelty was also one reason for the lack of credibility of charlatans and their nostrums among institutional practitioners: they had not the backing of tradition and textual authority.^72^ Unlike traditional remedies which, if prepared correctly, were already believed to work, the products perceived to require verification and additional licensing were those originating in heterodox quarters: chemical remedies, charlatans' secrets, and wonder drugs.^73^ The civic authorities, backed by the Colleges of Physicians and the Apothecaries' Arts, demanded trials for these preparations, which were carried out in liminal places: prisons, hospitals for the poor, and pesthouses.^74^ Testing for efficacy, in other words, was for the new and the noncorporative, and for those healers who lacked a supporting network in the city.^75^

While apothecaries were by no means dissociated from the culture of secrets, and indeed occasionally prepared remedies on behalf of empirics, it is telling that they rarely petitioned for licenses for proprietary remedies before the eighteenth century, and then did so mostly for the new genre of chemical remedies.^76^ The guild's oversight—from which charlatans and empirics were exempt—and the dynamics of a corporate economy should not be underestimated in relation to technical and pharmacological innovation.^77^ Exemplary, though not unique, is the case of Paolo Romani, Melichio's son-in-law and shop successor in Venice. In 1591 Romani[Other P-253] obtained a license for a new technique to make syrups in solid form, allegedly more palatable to the sick. His colleagues, however, objected to the privilege, and after two years of litigation, Romani was forced to share the recipe and revenue with the entire Venetian College of Apothecaries.^78^

This lack of incentives might also explain the scarcity of collections of secrets attributed to *speziali*. The handful that remain, notably the three volumes compiled by the Florentine Stefano Rosselli between the 1560s and the 1590s,^79^ support the view that thorough testing of recipes on the part of apothecaries occurred primarily for products tangential (cosmesis, cooking and confectionery, painting and dyeing) or extraneous (metallurgy, goldsmithing, decorative arts, chemiatria) to their trade. Rosselli's manuscripts gather hundreds of recipes for the above craft specialties—from tempering steel to baking sugar in the shape of books to counterfeiting gemstones—from sources of varying credibility. Similarly to how lay households dealt with extraneous knowledge in their recipe collections,^80^ they regularly employ the addendum *probatum est* (*provato*)—next to instructions for "confecting plums in the Genoese manner," for example, or an "ointment to stimulate venus to erection."^81^ Concerning mostly nontherapeutic products, the *prova*, the test, was meant to assess whether the recipe worked: namely whether one could manufacture the product from the instructions given.^82^ Occasionally, as the shop inventories testify, Rosselli followed this step with the decision to offer the product in his pharmacy at Saint Francis, mostly pastries and cakes, sold during weddings, funerals, and other festivities.^83^ This outcome should not be disjoined from the specific corporative reality of Florence, where, unlike other cities such as Venice, apothecaries still retailed all sorts of products besides medicines.^84^[Other P-254]

## The Language of Truth: From Sincerity to Authenticity

The advantage of adopting a skeptical stance toward "experimentation" as a driving force of practice in pharmacy shops is that we regain sight of the matters to which apothecaries actually accorded priority and visibility. Though possibly the most common "experimental action" in the workshop, tweaking was not singled out. It was one of the many tools utilized in the artisan's work of recipe exegesis, and had been there well before a discourse of experience and experimentation gained currency in the sixteenth century. Rather than actions, it is products to which Renaissance Italian apothecaries attached their reputation and public persona. These were not the most frequently produced, such as cheap purgatives and syrups,^85^ but those renowned for their efficacy and symbolic associations: poison antidotes like Theriac and Mithridate and complex compounds like Confection Alchermes prepared with scores of costly and rare imports from the eastern Mediterranean, the Levant, and India. These compounds were the only category of drugs, alongside poisons, to have been continuously regulated by guild and civic authorities since the thirteenth century and subjected to regular quality controls.^86^ They were the remedies that made the fortune and character of Italian pharmacy, attracting the scorn of self-styled reformers like Leonardo Fioravanti, at the same time as they transformed the pharmacy into a main site for the emerging culture of consumption.^87^

Going through the recipes for these preparations, we begin to notice an interesting phenomenon: the use of a language of sincerity and falsehood to describe and distinguish the ingredients prescribed for them. These consisted primarily of imports and the occasional indigenous plant that were laborious not only to procure but also identify. We are suddenly confronted with talk of "true apium,"^88^ "true rhaponticum,"^89^ "true euphorbium,"^90^ "true acorus," "legitimate cubeb,"^91^ "legitimate cardamom,"^92^ "false botrite,"^93^ "false balsam,"^94^ and so forth. In medieval[Other P-255] pharmacy sincerity had been invoked in a specific context, that of drug adulteration. Loudly condemned, this was commonly practiced by apothecaries to increase profits by using cheaper ingredients, diluting preparations, or increasing their weight by mixing them with foodstuffs of little therapeutic use.^95^ *Sincerus*, which, in the sense of unblemished and unmixed, had already been in classical usage,^96^ denoted something that had not been adulterated, and referred usually to a prepared product. In the sixteenth century the term lost currency, while retaining its meaning of something free of willful substitution or hampering.^97^ Thus, in his aptly named *Dialogue of the Deceptions of Certain Wicked Apothecaries* (1572), the Bergamasque doctor Giovanni Antonio Lodetto claimed that for every "good and sincere" box of manna sold in Rome, two had been "counterfeited" with sugar and starch.^98^

Increasingly, however, *sincerus* was supplanted in the literature by the terms "true" (Latin verus/Italian *vero*) and "legitimate" (*legitimus/legittimo*)—often used together in the coordinate "true and legitimate" (*verus ac legitimus*)—and pitted against "false" (*falsus/falso*) and "artificial" or man-made (*fictus/fattizio*). Importantly, this new taxonomy referred no longer to compounds but to *simplicia*, single ingredients. It concerned the source, not the process. The rather narrow notion of sincerity also shifted to embrace a wider definition that appealed to ontological, philological, and legal elements: "true" stood for authentic (the ingredient is what it proclaims and is proclaimed to be), correct (according to the ancients' original description), and licit (allowed by both texts and civic authorities). The language of sincerity and purity became one of authenticity, heralding a major taxonomic effort that introduced a new way of speaking, identifying, and thinking about materials in the Italian medical and botanical communities.

### A Genuine Nature

This quest for the genuine and the original was a conceptual novelty that reflected both internal developments in the field of pharmacy, and changing sensibilities in wider Italian society and culture. Regarding the[Other P-256] former, it was symptomatic of a crucial shift in emphasis and attention, which I see taking place in Renaissance pharmacy, from how remedies were made to what went in them. In the sixteenth century, ingredients regain center stage.

**Figure 3:**
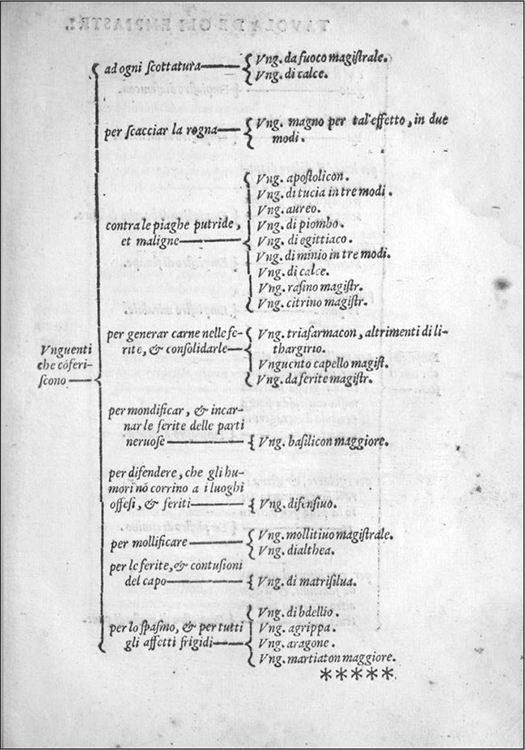
Table of medicaments from Girolamo Calestani, *Delle osservationi nel comporte gli antidoti e medicamenti che più si costumano in Italia all'uso della medicina* (Venice, 1562). Courtesy of the Wellcome Library, London.

In part, this development was the outcome of a discursive departure from the medieval pharmacology of degrees that had dominated the herbal and pharmacological literature well into the fifteenth century.^99^ As the new descriptive pharmacopoeias testify, by the mid-sixteenth century specific remedies had become associated with the treatment of specific complaints and diseases,^100^ and apothecaries no longer worried about painstakingly calculating the kind and degree of the humoral qualities of each ingredient that made up a compound. In other words, both therapeutic canon and preparation processes had been streamlined. An epigone of the first generation of apothecary authors, Giuseppe Santini, explained, "in [my manual] you'll see many compositions not at all dissimilar from those described by others, given that their ingredients' quantity and quality must not be altered."^101^ Rather than creating ad hominem preparations, apothecaries modified the product's overall dosage, ensuring it would not harm the patient when taken alongside the other remedies that completed the course of treatment, in line with the prevailing practice of polipharmacy. The descriptive pharmacopoeias supplied guidelines for matching remedies with ailments, as illustrated by the innovative set of tables introduced in Calestani's *Osservationi* (1562) and rapidly adopted by his peers (Figure 3). In these tables, basic tree diagrams link classes of remedies (e.g., syrups, purgatives, electuaries) to basic humors, conditions, and occasionally organ systems to treat (e.g., black bile or hot stomach). Standard named medicaments (e.g., Apostolicon Ointment) are also linked to defined ailments (e.g., festering sores).^102^ Probably this linear system had always been a necessity in practice, as the medieval tradition of *experimenta* testifies.^103^ In the sixteenth century, it found additional support in the new emphasis placed on communal over individual complexion,^104^ and in the popularity of ready-made secrets that promised to cure specific ailments whomever the sufferer.^105^[Other P-257]

[Other P-258]

The greatest push in the direction of the primacy of ingredients, however, came from the period's botanical renaissance. From the late fifteenth century, north Italian humanist botanists and their pupils devoted themselves to restoring the identity of the lost and mislabeled *materia medica* of the ancients through the tools of philology, fieldwork, and collecting. These efforts accompanied calls for renovation of the stock used in pharmacies, where confusion and errors were rife.^106^ As naturalists engaged in cross-referencing between texts and physical items, so the shop masters and their apprentices relearned to detect the "signs" and "true notes" of the contested *simplicia*. As the ingredients' identities were restored, the lost recipes of Galen and his venerable peers were returned to their "true form," in Filippo Costa's words.^107^ In practice, as we saw, this was often accomplished through tweaking. But this botanical revival also engendered in these artisans a new appreciation for nature as an object of study, and for its raw materials as specimens to be examined and preserved. Not only was this sentiment epitomized by the growing numbers of apothecaries collecting *naturalia* and seeking recognition in the naturalists' community, of whom Francesco Calzolari of Verona and Ferrante Imperato of Naples are the better known;^108^ an appreciation of nature over art, I argue elsewhere, may have been one reason for many Italian apothecaries' disinterest in chemical remedies and their radical transformation of materials before the 1620s.^109^ Indeed, it is for chemical substances like alum, vitriol, cinnabar, and sal ammoniac that the pharmacopoeias employ the term *fattizio* (artificial).^110^

These trends left their mark in the workshop. The quality of canonical recipes, as their success and failure, became primarily dependent on their ingredients rather than the techniques for their manipulation, consolidated after all by centuries of practice. The descriptive pharmacopoeias abound in remarks of the kind expressed by Borgarucci on Montagnana's Capital Cerate. Though used by most apothecaries, "some employ neither solid varnish, nor liquid styrax, possibly out of worry that the legitimate [ingredient] cannot be had."^111^ In turn, ensuring the ingredients' identity, especially of eastern *materia medica*, became a pressing concern, leading apothecaries to adopt a number of strategies of authentication. Additional[Other P-259] tests to verify that the rarer simple had not been mixed or substituted came to complement the routine sensory checks that its taste, smell, color, and weight conformed to its "archetype" and had not been weakened by age.^112^ For example, opopanax—the dried sap obtained from the root or stem of Dioscorides's Panax and often adulterated with wax—would be known as "sincere" if it "dissolve[d] like milk" when rubbed in water. False saffron would be given away by its color-not so light or able to stain one's tongue when chewed; tampered rhubarb by its taste-not at all astringent and with a slack texture, a clue that it was but some cooked stem.^113^ These tricks had been developed in the medieval period to combat the counterfeiting of gemstones and costly commodities; now they served to expose "vulgar costus" and other material impostors in the *spezierie*. Seeking external expertise became also increasingly common. In the 1590s the Mantuan master Antonio Bertioli sent an ampulla of balsam from Judaea for authentication to Prospero Alpino and Cortuso in Padua with a list of anxious questions, including whether true balsam always presented the two qualities of not staining woolen clothing and congealing milk, and whether balsam made artificially could mimic all characteristics of the true one. There is evidence of colleagues sharing particularly problematic ingredients once these had been ratified: it was from Bertioli that the Veronese Giovanni Pona procured verified opobalsam, carpobalsam, and xylobalsam for his Theriac.^114^'

**Figure 4:**
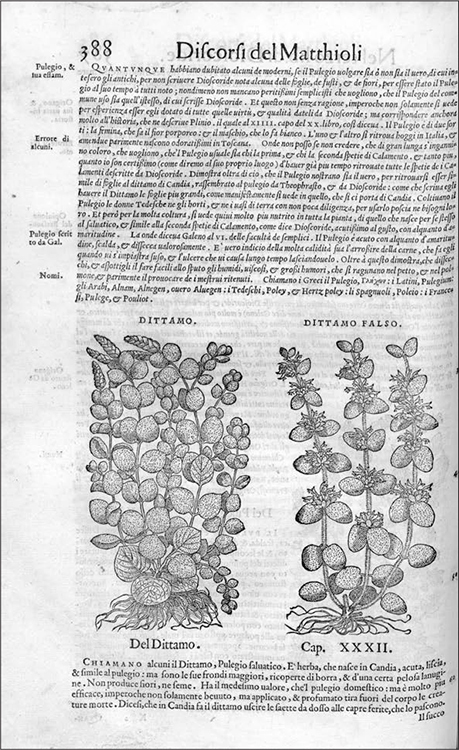
Comparative drawing of True Dictamus and False Dictamus, from Pietro Andrea Mattioli, *Discorsi nei sei libri di Pedacio Dioscoride Anazarbeo della materia médicinale* (Venice, 1559), 388. Courtesy of the Wellcome Library, London.

Collecting, a new development of the period, was similarly enlisted to help with learning and maintaining the distinctions between false and true. Apothecaries and physicians alike began to gather both correct and incorrect samples of theriacal and other medicinal ingredients, and display them side by side. The Venetian apothecary Marco Fenari owned the third variety of true costus.^115^ Calzolari's cabinet boasted "true balsam, true amomum, [true] costus, [true] folium, the truest aspalathus, [true] terra lemnia, the truest marble, [and] a most rare thing … true cinnamon."^116^ His Veronese colleague Giovanni Pona possessed twenty types of sealed earth (*terra sigillata*), some reputed true, some false.^117^ While "false" could[Other P-260] indicate adulterated, as often it described something very similar to the original but not quite the same. So, False Dictamus differed merely by its thicker leaves and milder aroma from True Dictamus, which, native to Crete, was also known as Dioscorides's First Dictamus (see Figure 4).^118^ A third variety was Vulgar or White Dictamus, so called because it could easily be found in Italy.^119^ Rather than an indictment, the label of false fulfilled a taxonomic function, contributing to classify known materials. For medical practitioners, these collections thus did not simply satisfy a taste for the curious and the exotic, but promised a very practical outcome: the creation of a canon of usable *materia medica*.

Yet, underlying this linguistic arrangement was also an important epistemological shift that permitted to regulate claims to knowledge and authority with new accuracy. Not only did these predicates of in/authenticity help stabilize the fluctuating language of medical botany.^120^ They also underscored the point that only the skillful could distinguish between true and false in nature. In the museum's pedagogical setting, a professor like Aldrovandi could use the display of trues and falses to teach his students—future practicing physicians—how to calibrate between deceivingly similar specimens that could threaten the integrity of the remedies they would prescribe, as well as alert them to the fraudulent substances circulating in the marketplace for drugs that they would eventually be called on to regulate. As the creator of this juxtaposition of truth and falsehood, the collector demonstrated to his peers his ability to discourse authoritatively about nature's materials.^121^ For the apothecary, the display (often located within or above the shop) had the additional advantage of showcasing his good will as far as his practice was concerned: his ingredients would be true, his compounds genuine. Such a claim would not have gone unnoticed in a marketplace where accusations of dishonesty were common.^122^[Other P-261]

[Other P-262]

### A Sincere Practitioner

Sincerity, as a quality denoting interiority, was, according to John Martin, invented in the Renaissance, and was tied to the period's seismic changes in understandings of individuality and the self.^123^ Sincerity stood in opposition to prudence, the cultivated ability to exercise caution but also dissimulate, in an age that contemporaries themselves described as one of plays on identity in the piazza, the court, and, especially, the church.^124^ Scholars have uncovered the growing anxiety of medieval and early modern Italian states to identify their subjects and travelers through their lands, and thus avoid the dangers of imposture and espionage. New ways were devised to prove that outward identity matched the person, notably through paperwork and visual stand-ins like printed portraits and passports.^125^ Intriguing parallels can be drawn with the new practice, among both apothecaries and naturalists, of producing written attestations (*fedi*) of an ingredient's authenticity—like that for aspalathus signed for Calzolari by the Patriarch of Aquileia and regularly exhibited to his museum visitors.^126^

But there is a deeper synergy between these developments and the new emphasis on authenticity in the pharmacy shop. Martin was elaborating Lionel Trilling's insight about the sixteenth-century transformation of sincerity into a moral category indicating a "congruence between avowal and actual feeling," a candid mirroring of the heart's intentions by outward speech and manners.^127^ Moral integrity was a quality consistently demanded of apothecaries in guild regulations, and in print by physicians and colleagues alike. By trading honestly, treating the poor charitably, and fearing God, "he will practice his art with all sincerity (*sincerità*) … profiting his family and rewarding his soul."^128^ Set against the backdrop of medical practice and the sale of drugs in the city, the ideal of transparency encoded in the newly found language of truth served the apothecary at[Other P-263] multiple levels. It verbalized a moral pact with the wider public, capitalizing on the economic impact that words possessed in a medical economy based on credit and on the reputations of supplier and customer, which hearsay could make and unmake.^129^ Its nomenclature also assuaged the concerns about rhetoric's role in medicine held by learned practitioners. While eloquence was thought to possess healing powers since antiquity, fifteenthand sixteenth-century medical authors had been expressing growing anxieties about the ethical role of mendacity and persuasion in cure.^130^ A new premium was placed on the doctor's directness of speech to his patient, devoid of embellishment and sophistication.^131^ As a rhetoric of spiritual honesty and commitment to the public good, this language of truth offered the framework for a renewed morality of the artisan and his products.

Yet, articulated through its terminology, I suggest, was not only the traditional professional and civic morality of not trespassing on patients' and peers' trust, but also the morality of staying "true to nature" (both nature sensed and nature read), the new ideal of Renaissance naturalists. Fighting dissimulation and insincerity, thus, was as much a professional imperative as a sign of intellectual probity and authority. If rhetorical "beliefs"—as Nancy Struever argues—generate "habits of action in inquiry,"^132^ this rhetoric of truth arguably fostered a research program: a return to the materials and a quest for the authentic. Linguistic sincerity and falsehood created a methodological circularity, sending artisans and naturalists alike to look for genuine items whether in storehouses or in fields and confirm the truth of those they already possessed, thus recapturing nature's original image.

Indeed, the elaboration of this language was almost certainly a collaborative effort between these two groups, the result of conversations that linked shops to university halls, and botanical gardens to scholars' libraries. Pinpointing its moment of birth remains difficult. Its terminology is noticeably absent from the philological works that set in motion the botanical renaissance. Theodorus Gaza's *Theophrasti De historia et causis plantarum* (1483), Ermolao Barbaro's *Castigationes Plinianae* (1492), and[Other P-264] Niccolò Leoniceno's *De Plinii erroribus* (1492) spoke of "lectio vera,"^133^ of returning the words of the ancients to their "verum sensum"^134^—not of a new class of in/authentic specimens. The more nuanced usage of true, false, and legitimate as adjectives locking the ingredient into a particular identity probably developed informally in the two following interrelated avenues.

In workshop practice, talk of true ingredients began to enter discussions of drug substitutes, notably around complex compounds like Theriac, in the early sixteenth century. Modeled on the Pseudo-Galen's *De succedaneis*, the established medieval genre of the *quid pro quo* had offered practical lists of ingredients that could replace materials that were rarer, costlier, or simply not on hand at the moment of need, without, however, explicitly addressing notions of an original simple or pinning a recipe to it. Indeed the substitutions were often bidirectional.^135^ The new literature of medical botany instead began to pit *succedaneum* against *verum*, introducing clearer suggestions of a qualitative hierarchy among ingredients.^136^ So Mattioli advised against using False Costus in place of the True because "notwithstanding it being beneficial, it is not as efficacious."^137^

However, it was through the epistolary exchanges of Italian and European botanists that the nomenclature fully solidified in the 1540s and 1550s, following the publication of the herbals and commentaries of Hyeronimus Bock, Leonhart Fuchs, Pietro Andrea Mattioli, and Rembert Dodoens, which provided a shared platform for the discussion on plant identification.^138^ The greatest input came from those overseeing or directly involved in remedy preparation, namely physicians, apothecaries, and the curators of the first botanical gardens. So we find Calzolari requesting Aldrovandi's opinion in a letter of 1561 "regarding [Dioscorides's] chapter on the squill, whether you believe the squills that we have to be the true ones or not."^139^ In 1558, Giovanni Fregoso of the *Eagle* in Padua had sent Aldrovandi "some Bolus armenus from Cyprus, with its false variety from Naples that was sold … at the time of plague at four and[Other P-265] five scudi per ounce."^140^ The terminology began to surface consistently in print in the 1560s in the interrelated genres of descriptive pharmacopoeias, essays on Theriac and Mithridate, and treatises on *res herbaria* in Latin and the vernacular.^141^ Not infrequently, as Mattioli's works testify, it was added from one edition to the next.^142^ By the 1590s, this nomenclature had become commonplace, and continued to be called upon in the seventeenth century whenever these rare and theriacal simples were discussed.^143^ Needless to say, by then it had also entrenched the existence of two tiers in the Italian apothecaries' perception of *materia medica:* the ancients' exotic ingredients and their compounds reigned over simpler remedies containing local plants identified by the terms *comune, volgare*^44^

### Theriac: Identity Stands for Efficacy

While of great symbolic resonance, the language of truth ultimately expressed the practitioners' anxiety at a material canon that had been enlarged (nominally at least) by the botanical revival and increased Mediterranean travel, yet whose products remained imperfectly known and difficult to acquire. The second edition of the *Ricettario Fiorentino* (1567), in no way exceptional, still contained asides of this sort: "Amomum is a plant today not known in Italy," and "the seed of this smaller siliqua may be used in place of the Greeks' true cardamom until that time when the true one is rediscovered."^145^ As Richard Palmer has argued and as the correspondence of Italian apothecaries involved in natural history testifies, such ingredients' reintroduction was slow and piecemeal, often limited to one individual pharmacy benefiting from one specific contact, whether a knowledgeable traveler or an enterprising collector.^146^ Ingredients like amomum and balsam remained controversial well into the seventeenth[Other P-266] century, prompting the flourishing from the 1590s of an interesting new genre of printed booklets that, between ten and one hundred pages, set out to disambiguate these simples celebrated by the ancients, survey all the existing literature pertaining to them, and explain their uses. Written by artisans and physicians alike, popular examples include the *Reasonings on Amomum and Aromatic Calamum* (1604) of the Venetian apothecary Cechino Martinelli Jr, *On the Balsam of the Ancients* (1623) of the Veronese Pona, and *De balsamo dialogus* (1591) of the Paduan professor and traveler through Egypt Prospero Alpino. Whether begun as a response to a colleague's rebuke, to a specimen observed on one's travels, or, in the case of Pona, to the discovery that Roman apothecaries composed their Theriac with false opobalsam "to the detriment of the truth and public good," these publications reveal the heightened concern for this subject among the healers of the peninsula.^147^

The relevance of these efforts in reading and disambiguation is nowhere clearer than in relation to the manufacture of Theriac and Mithridate, possibly the most iconic remedies in the Renaissance medical imaginary. The manner of their production best exemplifies how this idiom of truth, while reflecting an artisanal investment in botanical discourse, was also invoked by apothecaries to solve practical problems affecting their ability to commercialize certain products, and offered concrete returns in the marketplace. Its analysis closes the circle around our problematization of testing.

By the early modern period Theriac and Mithridate, born in antiquity as poison antidotes, were prized as the ultimate panaceas.^148^ For the apothecary their manufacture became an opportunity to display expertise and negotiate status before one's peers. Beside their therapeutic promise, the prestige conferred by their preparation lay not in the days of arduous labor it entailed (usually executed by hired hands), but in that it could be undertaken only after the successful sourcing of sixty to eighty ingredients. Among complex compounds, Theriac commanded the largest number of elusive *simplicia* originating from the East, including amomum, aspalathus, juice of acacia, aromatic calamus, costus, carpobalsamum, folius, marum, opobalsamum, seeds of thlaspi, terra lemnia, and petroselinum Macedonicum. While physicians insisted that only such original simples be used and decried the "abuse" of false ingredients, most remained on the desiderata list for the average master. Unsurprisingly, Theriac was[Other P-267] produced only once a year, and throughout the sixteenth century the race to create the perfect version of Andromachus and Galen was fought over the number of *succedanea* employed: from twenty to twelve to seven, until Calzolari allegedly triumphed by using only three.^149^

These substitutes were local plants and familiar imports with "almost all the notes" or virtues reputed comparable to those of the rarities prescribed.^150^ Juniper berries, for example, could take the place of carpobalsamum, almond oil that of opobalsam liquor, while the red earth Armenian bole could replace terra lemnia. Many of these substitutions had been worked out and standardized in the medieval period, and while *succedanea* were clearly assigned the role of maintaining the compound's overall authenticity, their choice was not left to the discretion of individual practitioners. The town medical authorities regularly issued printed lists of accepted substitutes and expected apothecaries to operate within their guidelines.^151^ The concern with legitimacy surfacing through the language of truth is linked to this regulated urban context, also evident in shop inspections.

The system allowed for some latitude, as it was expected that apothecaries would adapt their recipe in response to the availability of ingredients and *succedanea*, and to their guild's custom. Different towns produced different theriacs. Accordingly, rather than trying to enforce an unchanging script, the College of Apothecaries of Venice kept a reference collection of recipes for Theriac and Mithridate in use in Bologna, Florence, and elsewhere, and set out to approve the formula used by its local shops yearly.^152^ What took place in other shops was the object of much speculation among the artisans themselves. Inquiries were regularly made as to whether any *succedaneum* had been dispensed with, or a new ingredient found to be a more efficient substitute.^153^ This flexibility of the recipe—based on the number of *succedanea* employed, theriacs manufactured in the same town could contain different ingredients—seemingly had few consequences on the remedy's overall appreciation. Differences from one variety of the official compound to another were more epistemological[Other P-268] than therapeutic. Although the perfect compound was devoid of substitutes (and as such was never achieved in the sixteenth century), there was limited discussion of the potential inefficacy of theriacs containing a higher number of "replacements." Rather, anxiety concerned the product's age, as freshly made Theriac was considered weaker than the one that had been given years to mature.^154^

Nor was this effectiveness usually put to the test before selling the product. In *De Theriaca ad Pisonem*, Galen had provided instructions to determine whether Theriac worked upon maturation, and his advice was repeated by most medieval commentators.^155^ A lengthy version of the procedure still appeared in the much-consulted late fifteenth-century antidotary of apothecary Giuseppe Manlio, the *Luminare majus:* one should take a rooster or a pheasant and let it get bitten by a venomous animal, or place some venom in a small wound opened for this purpose between its wing and thigh. Once the rooster begins to weaken, it should be made to drink some Theriac with wine, and some should be poured on the wound. If the rooster lives, the Theriac is effective. Humans were spared this risk, but could be enlisted for an alternative test, which required the administration first of a purgative, then of a dose of Theriac. If the patient did not expel the compound, the latter could be considered effective.^156^

The learned tradition of poison trials was the main venue in which indirect testing of Theriac occurred.^157^ The antidote served to assay the potency of poisons in an exercise that involved primarily physicians, that is a group of university-educated practitioners who generally neither manufactured nor retailed the medicament.^158^ Instead, there is little evidence[Other P-269] that apothecaries tested their annual batches, obtained at such expense. Tellingly, the new descriptive pharmacopoeias generally omit Galen's instructions.^159^ When in the 1570s Aldrovandi tested his Theriac's efficacy on a rooster, this was probably a direct result of the dispute he was enveloped in. His recipe for the compound had been questioned by the Bolognese medical elite, who pitted it against the variety produced by the town's pharmacies and rejected it.^160^ There is evidence of experiences conducted, not on the compound itself, but on tangential issues concerning its ingredients, especially the famous vipers. Notably, the Neapolitan Ferrante Imperato staged the close observation of the vipers' parturition process, prompted by Galen's recommendation not to utilize pregnant ones.^161^ The extent to which these experiences were undertaken outside the group of apothecaries involved in natural history, however, should not be overestimated.

**Figure 5:**
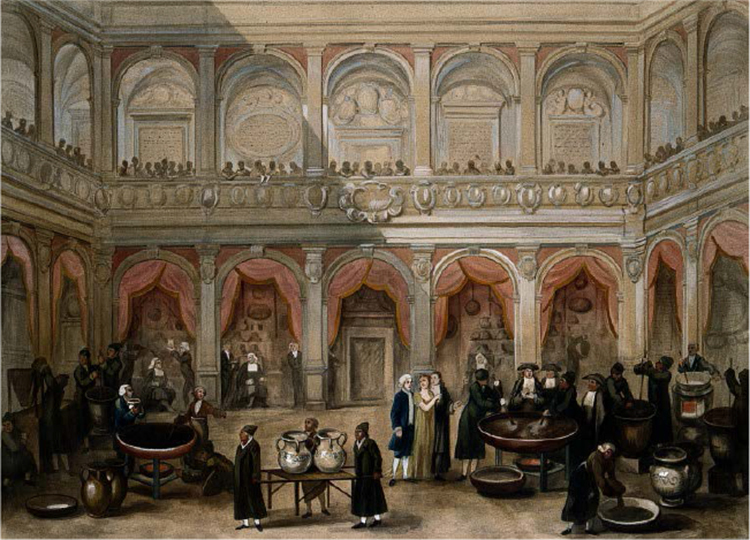
The manufacture of Theriac at Bologna. Gouache drawing by A. Terzi after Domenico Ramponi, 1818 (44599i). Courtesy of the Wellcome Library, London.

As a procedural step in the making and marketing of Theriac, testing would not have been a regular occurrence. In the sixteenth century, this open trial of Theriac and its ensuing spectacle were left to charlatans, who deftly staged their feigned poisoning and recovery.^162^ Conversely, apothecaries turned for validation to the product's public preparation (Figure 5).

Every year the antidote's manufacture proceeded according to a formalized routine. This began with a display over the shop's counter of the required simples, which were formally examined by representatives of the town's physicians and apothecaries for identity, freshness, and quality. Following their approval as "true and legitimate," the display was opened to the public, who were invited to scrutinize the master's newly proclaimed sincerity. Finally, after three days, the grinding and crushing began in large mortars placed on the street outside the pharmacy.^163^[Other P-270]

In this setting, the question of effectiveness is displaced. The ingredients' successful examination at the outset of the civic ceremony becomes sufficient proof of the worth of the remedy to come. Their truthfulness saves the apothecary from the need to test the overall product's efficacy. Indeed, the ability to source the authentic samples began to be crowned by *fedi* or attestations issued by the Physicians' Colleges and civic authorities. This proclamation of authenticity bolstered the artisan's reputation. He now had "fede amplissima" in Calzolari's words, "from my College and my city and the chancellors and rectors, and also from Messer Mattioli and many others."^164^ The *fede* was effectively a trademark that acknowledged a successful tweaking (here regarding the sourcing of ingredients)—only Calzolari could offer the three-substitute Theriac. While it did not confer monopoly, like patents for instruments the *fede* helped the artisan "live on" the products he devised.^165^[Other P-271]

Emboldened by these successes and in search of further visibility, from the 1550s it became customary for apothecaries to publish booklets recording their manufacture of Theriac and Mithridate in a particular year. Vendramino Menegacci of Vicenza immortalized his production of 1586, for example; Francesco Sartorio the one made in 1613 at the Bolognese Hospital of S. Maria della Morte; Giovanni Cardullo that of 1637 in Messina. The custom carried into the latter seventeenth century.^166^ These booklets provided a discussion raisonné of the recipe followed and of ingredients and *succedanea* used. They also strategically republished the attestations and privileges obtained, often ornamented by sonnets of praise and individual declarations by the physicians who had presided over the remedy's preparation. Complementing the genre of essays on rare *simplicia*, these booklets not only testify to the reflexivity in the practice of these artisans, but also restate where the core of anxieties and hopes of Italian apothecaries lay: in the dialogue with a long Mediterranean-based tradition that began with the Greeks and continued with their Arabic commentators, and in which apothecaries understood themselves to be rightful participants.

## Conclusion

If we wish to make sense of how sixteenth-century Italian apothecaries understood their work, we need to appreciate that their mode of validation, of proof and persuasion,^167^ was based not on notions of efficacy and novelty, but of authenticity. This explains the particular brand of testing examined and the values sustaining it.

Tweaking allowed the apothecary to remain faithful to the inherited tradition of pharmacology, both classical and medieval, while updating and upgrading it to make it viable under local circumstances of production. In doing so, it targeted the recipe's feasibility, not its reputed effects. Though the main experimental activity in the workshop, tweaking was reactive more than proactive. If it facilitated the introduction of changes in established workshop routines, it was not conceived as a source of radical innovation. In fact it reflected a different kind of deep chronology that apothecaries imagined for their art: one in dialogue with the past[Other P-272] rather than the future; one that saw advancement as restoration, rather than novelty production.

Expressing a new attention toward nature and its materials, the language of truth similarly enabled the reintegration of the ancients' *materia medica* into pharmaceutical practice. Its rhetoric was a currency that in different ways secured for the artisan a position of authority both in the marketplace and in the cabinets of curiosi. Its novel nomenclature restored confidence in the apothecary's trading practices by proclaiming the sincerity of his materials. It also sustained communication between artisan and learned, not only through the literature that flourished around it but also because it revolved around notions of loss, substitution, and recovery of originals that were central to many of the period's intellectual endeavors, from humanistic philology to antiquarianism.

This practical antiquarian disposition, and the corporative reality in which it was embedded, complicate any conventional image of artisanal epistemology that emphasizes tacit knowledge, hands-on learning, and boundless creativity. They offer an alternative model for how institutional pharmacy approached problems of validation and change, and succeeded in renewing its practice, a model that should be taken forward in any new analysis of the early modern medical trades.[Other P-273]

**** **For the supplementary appendix to this article click here.**

## Footnotes

1
SmithPamela H.The Body of the Artisan: Art and Experience in the Scientific RevolutionChicagoUniversity of Chicago Press2004

2
GentilcoreDavidMedical Charlatanism in Early Modern ItalyOxfordOxford University Press2006 RankinAlishaPanaceia's Daughters: Noblewomen as Healers in Early Modern GermanyChicagoUniversity of Chicago Press2013

3
BucklowSpikeImpossible RecipesSources and SerendipityHermensErmaTownsendJoyce H.LondonArchetype20091822 SmithPamela H.What Is a Secret? Secrets and Craft Knowledge in Early Modern EuropeSecrets and Knowledge in Medicine and Science, 1500–1800LeongElaineRankinAlishaAldershotAshgate20114766

4
ParkKatharineDoctors and Medicine in Early Renaissance FlorencePrincetonPrinceton University Press198512

5
EamonWilliamPharmaceutical Self-Fashioning or How to Get Rich and Famous in the Renaissance Medical MarketplacePharm. Hist453200312212915025072 GentilcoreMedical Charlatanism2209226

6
ShawJamesWelchEvelynMaking and Marketing Medicine in Renaissance FlorenceAmsterdamRodopi2011 PuglianoValentinaBotanical Artisans: Apothecaries and the Study of Nature in Venice and London, 1550–1610D.Phil. diss., Oxford University2012 WallisPatrickMedicines for London: The Trade, Regulation and Lifecycle of London Apothecaries, c.1610-c.1670D.Phil. diss., Oxford University20022 BenezetJean-PierrePharmacie et médicament en Méditerranée occidentale (XIII^e^-XVI^e^ sièclesParisHonoré Champion1999

7
KleinUrsulaSparyE. C.Materials and Expertise in Early Modern Europe: Between Market and LaboratoryChicagoUniversity of Chicago Press2010 RankinAlishaBecoming an Expert Practitioner: Court Experimentalism and the Medical Skills of Anna of Saxony (1532–1585)Isis981200723531753919910.1086/512830 KleinUrsulaThe Laboratory Challenge: Some Revisions of the Standard View of Early Modern ExperimentationIsis9942008769782

8
KleinUrsulaSparyE. C.IntroductionKleinSparyMaterials and Expertise7123

9
CookHarold J.The History of Medicine and the Scientific RevolutionIsis102120111021082166777810.1086/658659 WarnerJohn H.The History of Science and the Sciences of MedicineOsiris1019951641931161835410.1086/368748 LöwyIlanaHistoriography of Biomedicine: 'Bio,' 'Medicine,' and in BetweenIsis102120111161222166778010.1086/658661

10
SiraisiNancy G.Medieval and Early Renaissance Medicine: An Introduction to Knowledge and PracticeChicagoUniversity of Chicago Press1990

11
SchmittCharles B.Esperienza ed esperimento: un confronto tra Zabarella e il giovane GalileoFilosofia e scienza nel RinascimentoMilanLa Nuova Italia20012564

12
PuglianoBotanical Artisans63

13
OgilvieBrianThe Science of DescribingChicagoUniversity of Chicago Press2006

14
SchickoreJuttaTrying Again and Again: Multiple Repetitions in Early Modern Reports of Experiments on Snake BitesEarly Sci. Med152010567617

15
FindlenPaulaPossessing Nature: Museums, Collecting, and Scientific Culture in Early Modern ItalyBerkeleyUniversity of California Press1994194240 PuglianoBotanical Artisans64

16 Elaine Leong and I arrived independently at a cognate use of the concept of "tweaking." Leong uses the term to emphasize the open-ended nature of the recipes collected by lay households. For Leong, tweaking denotes the process whereby a recipe can be modified and reinvented each time it moves from one person to another (personal communication). In what follows, I offer a compatible but distinct definition of the practice of tweaking, one less open-ended and in use in a professional setting.

17
CalestaniGirolamoDelle osservationi nel comporre gli antidoti e medicamentiVeniceFrancesco Senese1562 BorgarucciProsperoLa fabrica degli spetialiVeniceVincenzo Valgrisi1566 MelichioGiorgioAvvertimenti nelle compositioni per uso della spetiariaVeniceGiovanni and Andrea Zenaro1575 CostaFilippoDiscorsi sopra le compositioni degli antidoti & medicamentiMantuaGiacomo Rufinelli1576

18
MinuzziSabrinaSul filo dei segreti medicinali: praticanti e professionisti del mercato della cura a Venezia (secoli XVI-XVIII)Ph.D. diss., University of Verona2012163

19
SantiniGiuseppeRicettario medicinaleVenice1604 FrancioniSalvatoreDe discorsi ne quali s'insegna l'Arte della spezieriaPalermo1625

20
BenezetPharmacie6116121 LongPamela O.Openness, Secrecy, Authorship: Technical Arts and the Culture of Knowledge from Antiquity to the RenaissanceBaltimoreJohns Hopkins University Press2001

21
CingolaniEnricoColapintoLeonardoDagli antidotari alle moderne farmacopeeRomeDi Renzo Editore2000 CorradiA.Le prime farmacopee italiane ed in particolare dei ricettari fiorentiniMilanRechiedei1887 StannardJerryHerbs and Herbalism in the Middle Ages and RenaissanceAldershotAshgate Variorum1999

22 Conversely, the selection excluded those remedies "appearing in Galen, Avicenna, Mesue and other ancient and modern authors … that have lost not only their usage but almost their very name among Apothecaries and Physicians" (Costa [n. 17], 23r-v).

23 Melichio (n. 17), 35v.

24 Ibid.

25 Costa (n. 17), 34v.

26 Borgarucci (n. 17), 559.

27 Melichio (n. 17), 18r.

28
SennettRichardThe CraftsmanNew Haven, Conn.Yale University Press2008179193

29 Costa (n. 17), 23v.

30
OgilvieScience of Describing13 SiraisiMedieval and Early Renaissance Medicine10

31
VosPaula DeThe 'Prince of Medicine': Yühanna ibn Masawayh and the Foundations of the Western Medical TraditionIsis104420136677122478349010.1086/674940

32 Calestani (n. 17), 261–62.

33 Costa (n. 17), 9v. The new pharmacopoeias—Borgarucci lamented—were themselves victims of the shoddy work of "little-alert printers, who, sometimes against their authors' very teaching and their proofreaders' excellent expertise, let such … important mistakes pass, which then cannot be remedied" (Borgarucci [n. 17], 441).

34 Calestani (n. 17), 125–26.

35
EpsteinStephan R.Transferring Technical Knowledge and Innovating in Europe, c. 1200–1800LSE Working Papers2005

36
SmithPamela H.Making Things: Techniques and Books in Early Modern EuropeEarly Modern Things: Objects and Their Histories, 1500–1800FindlenPaulaLondonRoutledge2013173203 SennettCraftsman28214238

37
RamplingJennifer M.Transmuting Sericon: Alchemy as Practical Hexegesis in Early Modern EnglandOsiris291201419342610374510.1086/678094

38
KristellerPaul O.Renaissance Thought II: Papers on Humanism and the ArtsNew YorkHarper1965

39 Costa (n. 17), 51r; Borgarucci (n. 17), 348.

40 Calestani (n. 17), Preface.

41
PomataGiannaObservation Rising: Birth of an Epistemic Genre, 1500–1650Histories of Scientific ObservationDastonLorraineLunbeckElizabethChicagoUniversity of Chicago Press20114580

42 The main exception was Florence's civic pharmacopoeia, the *RicettarioFiorentino*, whose first edition was printed in 1498. See the Online Appendix: http://muse.jhu.edu/resolve/19.

43
d'EremitaFra DonatoAntidotarlo … intorno … [ajll'elegere, preparare, componere e conservare i medicamenti semplici e compostiNaples1639132

44
MinuzziSul filo184546

45
BelloriniCristinaThe World of Plants in Renaissance Tuscany: Medicine and BotanyAldershotAshgate2016156157

46
MinuzziSul filo18137139 DallastaFedericaEredità di carta. Bibliotecheprivate e circolazione libraria nella Parma farnesiana (1545–1731MilanFranco Angeli2010244248272

47 They are consistently consulted, for example, by the Neapolitan apothecary and doctor Girolamo Donzelli, who authored both the official *Antidotario Napoletano* (1642) and the best-selling *Teatro farmaceutico, dogmatico e spagirico* (1675), e.g., 48 (Sciroppo di cicoria), 162 (Diagalanga di Mesuè), 276 (Trifera persica).

48 See Costa (n. 17), 23v, 34v, 51r.

49
EpsteinStephan R.Craft, Guilds, Apprenticeship, and Technological Change in Preindustrial EuropeJ. Econ. Hist5831998684711701

50 Melichio (n. 17), 120v.

51 Calestani (n. 17), 121.

52
McVaughMichael R.Arnaldi De Villanova Opera medica omnia, IIGranada and BarcelonaTobella1975330

53
EpsteinCraft, Guilds49 WallisMedicines for London6

54 Melichio (n. 17), 35v; Calestani (n. 17), 6, 98, 265.

55
PalmerRichardPharmacy in the Republic of Venice in the Sixteenth CenturyThe Medical Renaissance of the Sixteenth CenturyWearAndrewFrenchRoger K.LonieIain M.CambridgeCambridge University Press1985100117 WallisMedicines for London64

56 Costa (n. 17), 9r.

57 Melichio (n. 17), 21r. Costa also warns against "departing from the intention of the Authors" (Costa [n. 17], 23v).

58 Melichio (n. 17), 19r.

59
McVaughMichael R.The 'Experience-Based Medicine' of the Thirteenth CenturyEarly Sci. Med1420091051301983122710.1163/157338209x425524 VosDePrince of Medicine31687688

60
EdgertonDavidThe Shock of the Old: Technology and Global History since 1900LondonProfile2006

61
KleinSparyMaterials and Expertise713 RobertsLissaSchafferSimonDearPeterThe Mindful Hand: Inquiry and Invention from the Late Renaissance to Early IndustrializationAmsterdamKoninklijke Nederlandse Akademie van Wetenschappen2007

62
CookHistory of Medicine9

63
PalmerPharmacy55 CorradiLe prime farmacopee21

64
EpsteinCraft, Guilds49699701 AllenRobert C.Collective InventionJ. Econ. Behav. Org41983124 NuvolariAlessandroCollective Invention during the British Industrial Revolution: The Case of the Cornish Pumping EngineCambridge J. Econ2832004347363

65
RabierChristelleIntroduction: The Crafting of Medicine in the Early Industrial AgeTech. Cult543201343745910.1353/tech.2013.0090PMC417968027057070 GaudillièreJ.-P.Introduction: Drug TrajectoriesStud. Hist. Philos. Biol. Biomed. Sci3620056036111633755310.1016/j.shpsc.2005.09.005

66
KleinSparyMaterials and Expertise7125157 PuglianoValentinaThe Simple Alchemy of Renaissance ApothecariesThe Physician's Stone: Alchemy and Medicine from Antiquity to the EnlightenmentRamplingJennifer M.JonesPeter M.LondonRoutledge, forthcoming NewmanWilliam R.Promethean Ambitions: Alchemy and the Quest to Perfect NatureChicagoUniversity of Chicago Press2004

67
EdgertonDavidInnovation, Technology, or History: What is the Historiography of Technology About?Tech. & Cult51/32010680697

68 Borgarucci (n. 17), 678.

69
CriscianiChiaraHistory, Novelty and Progress in Scholastic MedicineOsiris619901181391161268410.1086/368697 McVaughMichael R.The Nature and Limits of Medical Certitude at Early Fourteenth-Century MontpellierOsiris6199062841161269110.1086/368695

70
BelloriniWorld of Plants45129153

71
Bellorini145

72
RankinAlishaEmpirics, Physicians, and Wonder Drugs in Early Modern Germany: The Case of the *Panacea Anwaldina*Early Sci. Med1420096807102050935710.1163/138374209x12542104913920 SiraisiNancyTheory, Experience and Customary Practice in the Medical Writings of Francisco SanchesBetween Text and Patient: The Medical Enterprise in Medieval and Early Modern EuropeGlazeF. E.NanceB. K.FlorenceSismel2011441463462

73
MinuzziSul filo1813

74
GentilcoreMedical Charlatanism2118149 Stevens-CrawshawJanePlague Hospitals: Public Health for the City in Early Modern VeniceFarnhamAshgate2012151182

75
GentilcoreMedical Charlatanism2119

76
MinuzziSul filo18133034

77
TrivellatoFrancescaGuilds, Technology and Economic Change in Early Modern VeniceGuilds, Innovation and the European Economy 1400–1800EpsteinStephan R.CambridgeCambridge University Press2008199231217

78 See note 76.

79
ZayasRodrigo deStefano Rosselli. Mes secrets: A Florence au temps des Médicis, 1593: patisserie, perfumerie, medicineParisJ.-M. Place1996

80
LeongElaineMaking Medicines in the Early Modern HouseholdBull. Hist. Med82120081451681834458810.1353/bhm.2008.0042

81
ZayasStefano Rosselli79187

82
SmithMaking Things36175

83
FlorenceMagistrato dei Pupilli del Principato27093v10v

84
ShawWelchMaking and Marketing6

85
ShawWelch237252

86 Compare the stability between the 1297 and 1565 statutes of the Apothecaries of Venice. Ugo Stefanutti, *Documentazioni cronologicheper la storia della medicina, chirurgia e farmacia a Venezia dal 1258 al1332* (Venice: Ongania, 1961), 43–48; Biblioteca Museo Correr, Venice, MS Cl.IV, 209/1, "Mariegola dei Spicieri," Ordini e capitoli, 29r.

87
WelchEvelynShopping in the Renaissance: Consumer Cultures in Italy, 1400—1600New Haven, Conn.Yale University Press2005

88 Calestani (n. 17), 42.

89 The Guild of Physicians and Apothecaries of Florence, *Ricettario Fiorentino* (Florence, 1574), 59.

90 Costa (n. 17), 39v.

91 Borgarucci (n. 17), 46, 26.

92
AnguillaraLuigiSemplicipareriVenice156121

93 *Ricettario* (n. 89), 72.

94 Melichio (n. 17), 16r.

95
WallisMedicines for London67

96
TrillingLionelSincerity and AuthenticityCambridge, Mass.Harvard University Press19721213

97 It often features in revised editions of medieval manuals, like the second *Ricettario* (n. 87 [1550]), e.g., 50–51, 52, 74.

98
LodettoGiovanni A.Dialogo de gl'inganni d'alcuni malvagi specialiPadua1626157222

99
McVaughArnaldi5231136

100 Present in Galen, this approach had receded in medieval literature (De Vos, "Prince of Medicine" [n. 31], 691–94).

101 Santini (n. 19), a2v.

102 Calestani (n. 17), [viiir-xviiv].

103
McVaughMichael R.The *Experimenta* of Arnald of VillanovaJ. Mediev. Renaiss. Stud11197110711711619667

104
StoreyTessaHealthy Living in Late Renaissance ItalyOxfordOxford University Press20133132

105
GentilcoreMedical Charlatanism2

106
OgilvieScience of Describing133049

107 Costa (n. 17), 51r.

108
PuglianoBotanical Artisans"6 EgmondFlorikeApothecaries as Experts and Brokers in the Sixteenth-Century Network of the Naturalist Carolus ClusiusHist. Universities2320085991

109
PuglianoSimple Alchemy66

110 *Ricettario* (n. 89), 14, 73, 129,

111 Borgarucci (n. 17), 872.

112
McVaughDetermining a Drug's Properties: Medieval Experimental Protocols18320910.1353/bhm.2017.002428757494

113 *Ricettario* (n. 89), 53, 59, 74.

114
BertioliAntonioIdea theriacaeMantua16025354 IdemBreue auuiso del vero balsamo, theriaca, et mithridatoMantua15965960

115 Biblioteca Universitaria Bologna (BUB), Adrovandi MS 136/v, 226v-227r, "Apud Dominum Marcum Fenarium Pharmacopolam."

116
CermenatiMarioFrancesco Calzolari da Verona e le sue lettere ad Ulisse AldrovandiRome191039

117
PonaGiovanniIndex multarum rerum quae repositorio suo adservanturVerona1601

118
PonaGiovanniMonte Baldo descrittoVenice161789

119
MattioliPietro AndreaDiscorsi nei sei libri di Pedacio Dioscoride Anazarbeo della materia medicinaleVenice1559390

120
PuglianoValentinaUlisse Aldrovandi's Color Sensibility: Natural History, Language and the Lay Color Practices of Renaissance VirtuosiEarly Sci. Med2020153583962685604810.1163/15733823-02046p04

121 I explore this further in "Fake Specimens in the Renaissance" in *The Matter of Mimesis*, ed. M. Bol and E. C. Spary (forthcoming).

122
GarzoniTommasoLa piazza universale di tutte leprofessioni del mondoVenice1587

123
MartinJohnInventing Sincerity, Refashioning Prudence: The Discovery of the Individual in Renaissance EuropeAmer. Hist. Rev1025199713091342

124
MartinJohnMyths of Renaissance IndividualismBasingstokePalgrave Macmillan2004103122 ZagorinPerezWays ofLying: Dissimulation, Persecution, and Conformity in Early Modern EuropeCambridge, Mass.Harvard University Press1990

125
GroebnerValentinWho Are You? Identification, Deception, and Surveillance in Early Modern EuropeNew YorkZone Books2007

126 BUB, Aldrovandi MS 136/v, 181r.

127
TrillingSincerity962

128
EpsteinSteven A.Wage Labour and Guilds in Medieval EuropeChapel HillUniversity of North Carolina Press1991155159

129
MuldrewCraigThe Economy of Obligation: The Culture of Credit and Social Relations in Early Modern EnglandNew YorkSt. Martin's199856

130
SchleinerWinfriedMedical Ethics in the RenaissanceWashington, D.C.Georgetown University Press1995548

131
McClureGeorge W.Healing Eloquence: Petrarch, Salutati, and the PhysiciansJ. Mediev. Renaiss. Stud152198531734611623454

132
StrueverNancy S.Rhetoric, Modality, ModernityChicagoUniversity of Chicago Press20093

133
BarbaroErmolaoCastigationes PlinianaeRome14923438

134
LeonicenusNicolausDe Plinii et aliorum medicorum erroribusFerrara150854

135
TouwaideAlain*Quidpro Quo:* Revisiting the Practice of Substitution in Ancient PharmacyHerbs and Healers from the Ancient Mediterranean through the Medieval WestArsdallAnne vanGrahamTimothyFarnhamAshgate20121961

136
CordusEuriciusBotanologiconCologne1534138

137 Mattioli, *Discorsi* (n. 119), 43.

138
OgilvieScience of Describing132837

139
CermenatiFrancesco Calzolari11635Calzolari to Aldrovandi261561

140 BUB, Aldrovandi MS 38/ii-iii, 219r (Fregoso to Aldrovandi, August 26, 1558).

141
AnguillaraSemplicipareri9262

142 Mattioli's first Latin edition of Dioscorides's *materia medica*, the *Commentarii* (Venice, 1554) makes occasional use of *legitimus* in the sense of "authentic." Its occurrence doubles in the 1559 edition. *Legitimo* features more sporadically in the illustrated vernacular *Discorsi* (Venice, 1557), but by the 1573 edition the full range of terms, particularly *vero*, is in operation.

143
DonatiAntonioTrattato de'semplici, pietre epesci marini che nascono nel lito di VenetiaVenice163138

145 *Ricettario* (n. 89), 17, 126.

146
PalmerPharmacy55

147
PonaGiovanniDel vero balsamo de gli antichiVenice16232

148
Nockels-FabbriChristianeTreating Medieval Plague: The Wonderful Virtues of TheriacEarly Sci. Med12320072472831817546310.1163/157338207x205115

149
CalzolariFrancescoLettera intorno ad alcune menzogne & calonnie date alla sua Theriaca da certo Scalcina PeruginoCremona1566 MarantaDella theriaca et mithridato libri duoVenice157234 QuattramiFriar EvangelistaTractatus perutilis atque necessarius ad Theriacam. Mithridaticamque antidotum componendamFerrara15975

150 *Ricettario* (n. 89), 18.

151 Ibid., 123–26.

152
ASVeGiustizia Vecchia211

153
CermenatiFrancesco Calzolari116134

154
MarantaDella theriaca149142148 Nockels-FabbriTreating Medieval Plague148

155 Avicenna and Mesue were the main conduit for Galenic thought on Theriac before the publication of Galen's *De Theriaca ad Pisonem* and *De Theriaca ad Pamphilium* in the 1530s, which together with Galen's *De Antidotis* became new reference texts. These circulated also in commentaries like Maranta's *De theriaca et mithridato libri duo* (Venice, 1572) and Orazio Guarguante's *Della Theriaca et sue mirabili virtu operetta* (appended to Melichio [n. 17]).

156
ManlioG. G.Luminare maggioreVenice155980v81r

157
PastoreAlessandroIl trattato De venenis e la tradizione tossicologica del suo tempoGirolamo Mercuriale: Medicina e cultura nell'Europa del CinquecentoArcangeliAlessandrNuttonVivianFlorenceLeo S. Olschki2008233246

158 The apothecaries' testing of the few poisonous substances they had been allowed to retail since the thirteenth century (e.g., chanterelle mushrooms, opium, sublimate, and arsenic) seems, by the sixteenth century, to have been infrequent. Similarly, although the botanical renaissance raised concerns over fatal mistakes in the identification of new and old ingredients, trials of disambiguation (usually by feeding such ingredients to dogs) were not systematic. Instead, they seem to have involved primarily apothecaries like Calzolari already fully invested in the study of nature, and to have crystallized around a few problematic items debated in the new literature on *res herbaria*, notably poison nut (*nux vomica), doronicum*, and *aconitum*. See Cermenati, *Francesco Calzolari* (n. 116), 59 (Calzolari to Aldrovandi [1595–96?]); Borgarucci (n. 17), 400.

159 They feature only in Calestani (n. 17), 91ff.

160
OlmiGiuseppeFarmacopea antica e medicina moderna. La disputa sulla teriaca nel Cinquecento bolognesePhysis19197719824611630866

161
StendardoEnricaFerrante Imperato: Collezionismo e studio della natura a Napoli tra Cinque e SeicentoNaplesAccademia Pontaniana2001134136

162
GentilcoreMedical Charlatanism2174

163
MenegacciVendraminoLa Theriaca et il Mithridato composti in VicenzaVicenza1587A2v CardulloGiovanniTheriaca d'Andromaco compostapubblicamente in MessinaMessina163789 StösslMarianneLo spettacolo della Triaca. Produzione e promozione della 'droga divina' a Venezia dal Cinque al SettecentoQuaderni Centro Tedesco Studi Veneziani251983447

164
CermenatiFrancesco Calzolari11639

165
BiagioliMarioFrom Print to Patents: Living on Instruments in Early Modern EuropeHist. Sci442006139186

166
MenegacciLa Theriaca163 SartorioFrancescoDiscorso sopra la compositions della triaca da lui composta in Bologna a 15 agosto 1612Bologna1613 CardulloTheriaca162

167
SerjeantsonRichardProof and PersuasionThe Cambridge History of Science3DastonLorraineParkKatharineCambridgeCambridge University Press2007132176

