# Supplementary material for: Pharmacy, Testing, and the Language of Truth in Renaissance Italy
Source: Bull Hist Med. 2017 Summer;91(2):233-273. doi: 10.1353/bhm.2017.0026 (PMC5663472; doi:10.1353/bhm.2017.0026)
Supplement: Supplementary file 1 [file bhm-91-2-233_supp01.pdf]

Pharmacy, Testing, and the Language of Truth in Renaissance Italy

VALENTINA PUGLIANO

ONLINE APPENDIX: ITALIAN APOTHECARIES' TRADE LITERATURE IN PRINT 1500-1700

Predecessors

Giovanni Giacomo Manlio, *Luminare majius super Mesue antidotarium et practicam* (Pavia: Antonio De Carcano, 1494).

Paolo Suardo, *Thesaurus aromatariorum* (Milan, 1496); (Lyon, 1536).

Descriptive pharmacopoeias – First generation

Girolamo Calestani [Parma], *Delle osservationi nel comporre gli antidoti e medicamenti che più si costumano in Italia all'uso della medicina* (Venice: Francesco Franceschi Senese, 1562; 1564; 1568; 1570; 1575; 1580; 1584; 1589; 1597; 1606; 1616; 1623; 1655; 1673; 1677).

Prospero Borgarucci [Padua], *La fabrica degli spetiali* (Venice: Vincenzo Valgrisi, 1566; 1567).

Giovanni Pietro Cerasio [Rome], *Methodo dello spetiale nel quale si tratta del vero modo di perfettamente formare qual si voglia composto medicinale* (Rome: Heirs Antonio Baldo, 1574); (Milan, 1611).

Giorgio Melichio [Venice], Avvertimenti nelle compositioni per uso della spetiaria Con vna diligente esaminatione di molti simplici, tratta da piu degni auttori antichi et moderni (Venice: Giovanni and Andrea Zenaro, 1575); (Venice, 1596, 1605 edited by Paolo Romani); (Venice, 1627; 1648;

1660; 1671; 1678; 1688; 1720 edited by Alberto Stecchini); (1667; 1682 edited by Antonio De Sgobbis as Universale Theatro farmaceutico).

Giorgio Melichio and Samuel Keller, *De recta medicamentorum, quorum hodie usus est, parandorum ratione commentarij… in Latinum sermonem conversi a Samuel Keller* (Wittenberg: Johannes Crato, 1586).

Filippo Costa [Mantua], *Discorsi sopra le compositioni degli antidoti & medicamenti che più si costumano di dar per bocca* (Mantua: Giacomo Ruffinelli, 1576; 1586).

Descriptive pharmacopoeias – Epigones:

Giuseppe Santini, *Ricettario medicinale di M. Giuseppe Santini Lucchese Speciale al Corallo* (Venice: Marco Claseri, 1604).

Salvatore Francioni, *De Discorsi libri tre ne' quali s'insegna con diligenza a discepoli dell'arte, l'arte della spezieria* (Palermo: Antonio de Franceschi, 1625).

Fra Donato D'Eremita Dell'Ordine dei Predicatori, *Antidotario nel quale si discorre intorno all'osservanza che deve tenere lo spetiale nell'elegere, preparare, componere e conservare i medicamenti semplici e composti* (Naples: Secondino Roncagliolo, 1639).

Giuseppe Donzelli, *Teatro Farmaceutico, Dogmatico e Spagirico nel quale s'insegnano una molteplicita' di arcani chimici, piu' sperimentati dell'autore. Aggiuntovi a contemplatione de i pij e divoti professori un catalogo de i santi medici* (Naples: Giacinto Passaro, 1667; 1675; 1726); (Rome, 1677); (Venice, 1681; 1686; 1696; 1704; 1713; 1728).

Francesco Sirena [apothecary of the Franciscan convent of Santa Croce in Pavia], *L'arte dello spetiale* (Pavia: Giovanni Ghidini, 1679); (Venice, 1680).

Civic pharmacopoeias

College of Physicians of Florence, *Ricettario Fiorentino* (Florence: Compagnia del Drago, 1498).

College of Physicians and Guild of Apothecaries of Florence, *El ricettario dell'Arte, et vniuersita de medici, et spetiali della città di Firenze. Riueduto dal Collegio de Medici per ordine dello illustrissimo et eccellentissimo signore duca di*

Firenze (Florence: Lorenzo Torrentino, 1550; 1562; Giunti, 1567; 1574; 1597; 1623; 1670; 1696).

College of Physicians of Mantua, *Antidotarium Mantuanum ex multis optimisque authoribus collectum, castigatum et accurate digestum* (Venice: Vincenzo Valgrisi, 1559).

Ulisse Aldrovandi and College of Physicians of Bologna, *Antidotarii Bononiensis sive de usitata ratione componendorum, miscendorumque medicamentorum epitome* (Bologna: Giovanni Rossi, 1574; 1606).

College of Physicians of Bergamo, *La farmacopea o' antidotario dell' eccellentissimo Collegio de' signori medici de Bergomo nel quale si contiene il modo di comporre i medicamenti hoggidì più usitati nelle spetiarie* (Venice: Niccolò Moretti, 1597; 1628).

Ippolito Ceccarelli and College of Physicians of Rome, *Antidotario Romano* (Rome: B. Zannetti, 1612); (Rome: Giacomo Ruffinelli, 1624); (Milan, 1635; 1637 with commentary by Pietro Castelli).

Curzio Marinelli, *Pharmacopaea, sive De vera pharmaca conficiendi et praeparandi methodo a Medicorum Venetorum Collegio Comprobata, libri duo* (Venice: Roberto Meietto, 1617) – withdrawn from the market.

Giovanni Battista Cortesi, *Pharmacopoeia seu Antidotarium Messanense, in quo tum simplicia tum composita medicamenta, vsu recepta accurate examinantur* (Messina: Pietro Brea, 1629).

Giuseppe Donzelli and College of Apothecaries of Naples, *Antidotario napolitano di nuouo riformato, e corretto dall'almo Collegio de Spetiali... Oue si contengono tutte le ricette delli medicamenti, tanto semplici, quanto composti, che necessariamente deuono tenere, e mostrare nelle regie visite, tutti li spetiali di questa fidelissima città, e regno. Con vtilissime, e fruttuose annotationi di Giuseppe Donzelli* (Naples: Francesco Savio, 1642; 1649; 1653).

Apothecaries' booklets on their theriac and mithridate

Francesco Calzolari, *Lettera intorno ad alcune menzogne & calonnie date alla sua Theriaca da certo Scalcina Perugino* (Cremona: Vincenzo Conti, 1566).

Pastarino, *Ragionamento sopra l'Arte della Speciaria. Alli Magnifici et Illustri Senatori di Bologna acciocche' si piglino cura di questo utilissimo essercitio* (Bologna: Giovanni Rossi, 1575).

Giovanni Francesco Lauro, *Lode della theriaca d'Andromaco fatta in casa del M. Giulio Affaruosi spetiale in Reggio* (Reggio [Emilia]: Ercoliano Bartoli, 1578).

Vendramino Menegacci, *La Theriaca et il Mithridato composti in Vicenza per Vendramino Menegacci speciale alla Fortuna* (Vicenza: Perin and Greco, 1587).

Antonio Bertioli, *Breue auuiso del vero balsamo, theriaca, et mithridato. Vltimamente composti a commune beneficio per li fratelli Berthioli spetiali* (Mantua: Francesco Osanna, 1596).

Antonio Bertioli, *Idea Theriacae, et Mithridatii, ex optima, atque omnium excellentissima Antonii Berthioli Pragmatia. Ipsorumque interim ingredientium simplicium exactissima discussio, et praesertim de viperis scitu dignissima; non hucusque ab alio quopiam Physiologo enucleata, nunc demum a Iacobo Ferrario philosopho, & medico Mantuano, partim ex doctissimi olim Flamminii Evoli scriptis, partim ex proprijs excerpta, & ad commune commodum edita* (Venice: Antonio and Jacopo De Franceschi, 1601); (Mantua: Francesco Osanna, 1602).

[Adriano Riccardi], *Breve istruttione introno al comporre la theriaca d'Andromaco. Di nuovo raccolta da Adriano Riccardi. Al Sig. Antonio Bertioli meritissimo special del Serenissimo di Mantova* (Bologna: Vittorio Benacci, 1606).

Francesco Sartorio, *Discorso sopra la compositione della triaca da lui composta secondo la ricetta di Andromaco il Vecchio nell'Hospitale di Santa Maria della Morte in Bologna a 15 agosto 1612* (Bologna: Vittorio Benacci, 1613).

Ottavio Campolongo, *Considerationi di Ottavio Campolongo parmegiano spetiale in Vinegia all'insegna del Forno intorno alla theriaca: dove si scroprono secondo l'opinione di Galeno e d'altri celebri scrittori molti gravissimi errori finhora commessi da coloro che la compongono* (Venice: G.B. Bertoni, 1614).

Asdrubale Mostravero, *Risposta alle Considerationi d'Ottavio Campolongo Parmegiano, speciale in Venetia all'insegna del Forno, intorno alla compositione della teriaca, dove si mostra come quella è stata sempre legalissimamente composta,*

& si scopre la vanità de'pretesi & annoverati errori da lui, composta per mano di Asdrubale Mostravero, già speciale in Milano & hora publico professore di matematica (Ravenna: Stampatori Camerali, 1614).

Baldassarre and Michele Campi, *Nuouo discorso col quale si dimostra qual sia il vero mitridato. Contra l'antichissima opinione di tutti gli scrittori, e contra il commun costume di tutti gli aromatarij. Con vn breue capitolo del vero aspalato* (Lucca: Ottaviano Guidoboni, 1623).

Giovanni Domenico Cardullo, *Theriaca d'Andromaco composta pubblicamente in Messina da G.D. Cardullo Messinese spetiale all'insegna di S. Giorgio ove tutti li semplici di quest'antidoto sottilmente s'esaminano, si dichiarano e s'approvano* (Messina, 1637).

Alessandro Castelli, *Dell'uso et virtù della theriaca di Andromacho il vecchio, composta et dispensata per noi Alessandro e Giorgio Castelli specieri alla Madonna in S. Bortolamio in Vinetia* (Venice: Heirs of Gio. Salis, 1650).

Giovanni Francesco Savaro, *Relazione dell'apparato nella mostra della Theriaca e del Diacinto, esposta... nella speziaria della Regina Mora di Roma a 28, 29 e 30 di giugno... 1652* (Rome: F. Moneta, 1652).

Giuseppe Candrini, *Teriaca d'Andromaco e Mitridato di Democrate composta da me Giuseppe Candrini* (Modena: Viviano Soliani, 1677).

Antonio Fontaniva, *Dell'uso et virtù della Theriaca di Andromaco il Vecchio composta et dispensata per me Antonio Fontaniva* (Venice: [na], 1682).

Apothecaries' short essays on individual theriacal ingredients

Cechino Martinelli, *Ragionamenti sopra l'amomo et calamo aromatico. Nuouamente l'anno 1604 hauuto di Malaca città d'India dall'eccell. sig. Cechino Martinello suo zio* (Venice: Gratioso Perchacino, 1604).

Bernardino Vecoli, *Della preparatione della pietra lazzoli per la confettione Alchermes. Al molto illustre uffizio de Signori Provveditori sopra l'Arte della Spetiaria. Con alcune considerationi di Nicolo' Mazza e di Herofilo Serafini* (Lucca: Ottavio Guidoboni, 1617).

Giovanni Pona, *Del vero balsamo de gli antichi commentario sopra l'Historia di Dioscoride nel quale si proua, che solo l'opobalsamo arabico è il legitimo; e s'esclude*

ogn'altro licore, abbracciato sotto nome di balsamo degli antidoti (Venice: Roberto Meietti, 1623).

Baldassarre and Michele Campi, *Parere sopra il balsamo di Baldassarre e Michele Campi aromatari peritissimi* (Lucca: Pellegrino Bidelli, 1639).

Baldassarre Campi, *Al Sig. Antonio Manfredi aromatario diligentissimo in Roma. In risposta ad alcune obiettioni fatte nel libro nostro del balsamo* (Lucca: Pellegrino Bidelli, 1640).

Baldassarre and Michele Campi, *Al sig. Antonio Manfredi aromatario diligentiss. in Roma Indilucidotione, e confermatione maggiore di alcune cose state da noi dette nella risposta al sig. Gaspari medico in Roma* (Pisa: Francesco delle Dote, 1641).

Giuseppe Donzelli, *Parere dell'almo Collegio de' Spetiali di Napoli sopra l'opobalsamo mandatoli dalli signori console del Collegio de' Spetiali di Roma. Con un picciolo trattato dell'opobalsamo orientale di Gioseppe Donzelli* (Naples: Francesco Savio, 1640).

Giuseppe Donzelli, *Lettera familiare sopra l'opobalsamo orientale adoperato in Roma dalli signori Antonio Manfredi e Vincenzo Panuzzi in far le lor teriache* (Padua: Paolo Frambotti, 1643).

Trivulso Giaquinto, *Ragguaglio primo venuto di Parnaso l'anno MDCXXXX sopra il balsamo d'Arabia* (Trent: Santo Zanetti, 1640).

Baldassarre and Michele Campi, *Spicilegio botanico discorso fatto in dialogo di Baldasar, e Michele Campi aromatari di Lucca, nel quale si manifesta lo sconosciuto cinnamomo delli antichi, si mettono in chiaro alcuni semplici di oscura notitia, et alcuni del tutto nuoui alla luce si espongono. Opera curiosa, & vtile per chimici, & spetiali* (Lucca: Iacinto Paci, 1654, 1669).

Physicians' short essays on individual theriacal ingredients

Prospero Alpino, *De balsamo dialogus in quo verissima balsami plantae, opobalsami, carpobalsami, et xilobalsami cognition plerisque antiquorum atque iuniorum medicorum occulta nunc elucescit* (Venice: Paolo Frambotto, 1591).

Giovanni Falcone, *De balsamo ad Perillustrem, & Reuerendiss. DD. Aurelium Pomponatium prothonot. apostolicum... Carmen* (Mantua: Francesco Osanna, 1596).

[Anonymous], *Giudicio sopra i ragionamenti di Cecchino Martinelli sopra il nuovo amomo et calamo aromatico di Malacca d'India d'incerto auttore* (Mantua: Francesco Osanna, 1605).

Nicolò Marogna, *Commentarius in tractatus Dioscoridis et Plinii de amomo* (Basel: Lazari Zetzneri, 1608); also translated into Italian and appended to Giovanni Pona, *Monte Baldo descritto* (Venice: Roberto Meietti, 1617).

[Giovanni Nardi and Johan Vesling], *Francesco Panuzzi romano a i lettori. Eccovi ò lettori per commune intelligenza la traduttione di latino in volgare di due lettere sopra il balsamo* (Rome: Ludovico Grignani, 1640).

Francesco Perla, *De orientali opobalsamo nupèr in theriacae confectione adhibito, et inter Romanos medicos controuerso, historica & physica dissertatio* (Rome: Ludovico Grignani, 1641).
